# Towards a better understanding of work participation among employees with common mental health problems: a systematic realist review

**DOI:** 10.5271/sjweh.4005

**Published:** 2022-03-31

**Authors:** Suzanne GM van Hees, Bouwine E Carlier, Emma Vossen, Roland WB Blonk, Shirley Oomens

**Affiliations:** 1 Occupation and health research group, HAN University of Applied Sciences, Nijmegen, The Netherlands; 2 Department Tranzo, Tilburg School of Social and Behavioral Sciences, Tilburg University, Tilburg, the Netherlands; 3 Department Human Resource Studies, Tilburg School of Social and Behavioral Sciences, Tilburg University, Tilburg, the Netherlands; 4 TNO, Leiden, The Netherlands; 5 Optentia, North West University, Vanderbijlpark, South Africa; 6 Radboudumc, Department of Primary and Community Care, Nijmegen School of Occupational Health

**Keywords:** capability approach, mental health, realist research, stay at work, sustainable employment, work ability

## Abstract

**Objectives:**

Common mental health problems (CMHP) represent a major health issue and burden to employees and employers. Under certain conditions work contributes to wellbeing and participation of employees with CMHP. Promoting work participation is important, however the specific conditions in which work participation occurs is complex and largely unclear. This calls for a novel, realistic approach to unravel the complex relationship between outcomes, context and underlying mechanisms of work participation.

**Methods:**

In the present realist review, peer-reviewed studies conducted between 1995 and 2020 were systematically reviewed on the outcome measures ’stay at work’ (SAW) and ’work performance’ (WP). The database search from seven databases identified 2235 records, of which 61 studies met the selection criteria and methodological rigor.

**Results:**

The synthesis demonstrates how work participation is promoted by the following mechanisms and contextual factors: (i) organizational climate and leadership, (ii) social support, (iii) perceived job characteristics, (iv) coping styles, (v) health symptoms and severity, (vi) personal characteristics, and (vii) features of interventions. An explanatory framework, based on the Capability-for-Work model, presents a new set of capabilities leading to SAW and WP.

**Conclusions:**

This systematic realist review revealed mechanisms and contextual factors that promote both SAW and WP among employees with CMHP. These show how the organizational climate, social support in the work context, job characteristics and certain capabilities enable employees with CMHP to participate at work. Our contributions and practical implications are discussed, providing valuable insights for employers, professionals and researchers in the development of evidence-based interventions.

Work participation among employees with common mental health problems (CMHP) is an increasingly important, yet highly complex phenomenon (1). The complexity of work participation is that work can cause CMHP and on the contrary, it can be the solution to those who are affected by CMHP. Under certain conditions, work contributes to the well-being and work participation of employees with CMHP. As the Organisation for Economic Co-operation and Development (OECD) calls for preventing instead of reacting to negative work outcomes, such as sickness absence and reduced work capacity (2), a thorough understanding of how to promote work participation is needed. Common mental disorders refer to depression, anxiety disorder, or stress-related disorder (3, 4). However, a large number of employees who suffer from such problems are undiagnosed and do not receive treatment (5). We also consider this group of employees at risk of negative work outcomes, as a consequence of psychological complaints. Since most people affected by CHMP or psychological complaints are employed and actually working, this phase needs increased attention while the individual is at work (6, 7). Therefore, we use a relatively broad definition of employees with diagnosed mood, anxiety or stress-related problems as well as self-reported psychological complaints.

Previous studies on work participation among employees with CMHP show that staying at work and being productive is affected by individual factors such as higher symptom severity (eg, a past history of CMHP, co-morbidity), and work-related factors (eg, lower job control, job strain or a supportive work environment) (1, 8). While these studies give an insight into factors that promote or hinder work participation, it remains unclear what really enables employees with CMHP to effectively continue working? As work participation is both a means and a goal to promote one’s level of work performance and the ability to stay at work, we need to unravel these two aspects and how they interact in order to develop effective interventions for employers (8, 9). Other reviews in occupational health have concluded that the interaction between work outcomes, the underlying mechanisms, and how actors in the work environment collaborate have proven crucial to intervene effectively, and yet these are not yet clearly understood (5, 10, 11). Therefore the present study addresses the recommendation to move from ‘what works’ to promote work participation to ‘what works, for whom, under what circumstances and how’ (12, 13). This calls for a novel approach in our attempt to understand work participation, in which realist research may provide a suitable methodological answer. Pawson et al (14) developed the realist review approach from the philosophical tradition of critical realism, which seeks to consider the complexity of causal relations when explaining social interactions and interventions (9, 14). It is a theory-driven evaluation method providing an analysis that is more explanatory in nature.

Our initial program theory to develop an explanatory framework for work participation is the Capability-for-Work model (15). This model is based on the concept of capability, as developed by Sen (16). Capabilities represent a person’s opportunity and ability to achieve certain human functionings, taking into account someone’s particular circumstances. Previous articles have applied the literature on human development and capabilities to the work context (17, 18). Among the many things that human beings might develop the capacity to do, employment and work are addressed as a functioning (19). Furthermore, following Sen (16), it is not enough to establish the resources individuals have, but rather to consider what they can actually do or become with those resources to achieve certain (work) functionings. These so called ‘conversion factors’ refer to the process of converting one’s resources to tangible capabilities, resulting in work functioning that the employee chooses to achieve. In this, Bonvin (20) refers to personal and social conversion factors, which play a key role with regard to capability for work.

In this study, work participation is operationalized by two work outcomes (21). The first outcome is stay at work (SAW), that is, *‘the employee is currently working’* addressing a relatively new concept in the field of occupational health that has no uniform definition in the literature (22). We define SAW as continuing to work, indicated as no absenteeism or not being absent >50% or ≤6 weeks (8, 23). Besides SAW, we are interested in different facilitators of work performance (WP), or *‘how the employee functions at work’*. WP refers in the present review to subjective (self- or other rated) performance or objective (externally rated) performance (24). Derived from the Capability-for-work model, we hypothesized that work participation is determined by the way an employee succeeds in converting personal- and work inputs and resources (ie, conversion factors) into capabilities and subsequently into work functioning such as SAW and WP (15).

To the best of our knowledge, a realist synthesis of evidence relating to SAW and WP for employees with CMHP has not been conducted thus far. In this study, we aim to create a better understanding of work participation by providing a robust, systematic overview of current knowledge and by developing an explanatory framework. To do so, this study adopts a systematic realist review approach. The following research question guided this approach: What mechanisms promote SAW and WP (work outcomes), for whom, under what circumstances and how, amongst employees with CMHP?

## Methods

### Identification and selection process

For the sake of readability, in this section we briefly report the steps followed in the review process. A more detailed description of the review methodology is provided in the supplementary material, www.sjweh.fi/article/4005, Appendix A, including the identification and selection process, use of theory and appraisal tools, and data extraction and synthesis. The systematic realist review followed the steps and procedures outlined by RAMESES publication Standards for Realist Synthesis (25). Details of the protocol for this systematic realist review are registered on PROSPERO and can be accessed at www.crd.york.ac.uk/prospero/display_record.php?RecordID=108913 and can be found in the published study protocol (21). Regarding the search strategy and study selection, we adhered to the PRISMA guidelines for the conduct of systematic reviews (26). All scientific peer-reviewed studies available between 1 January 1995 and 26 June 2020 were retrieved in this systematic realist review. We conducted a computer-based search in the following databases, Pubmed, Medline, PsycInfo, Embase, Cochrane, Cinahl and Web of Science. An example can be found in supplementary Appendix A. Three independent authors dually assessed the studies’ rigor and relevance in each of the following phases using the selection criteria (table 1): title and abstract screening, full text screening and quality appraisal using the Mixed Methods Appraisal Tool (MMAT) (27) and data extraction.

**Table 1:** Inclusion and exclusion criteria.

Inclusion criteria	Exclusion criteria
Primary outcome: stay at work, absence of absenteeism, continue working, being at work – subjects had to perform paid work, either part or fulltime. If recorded as sick, subjects had to work for ≥50% within the first 6 weeks after their first sick day.	Studies including a general population of workers, and their mental health or workers targeted in primary stress prevention (not providing subgroups with workers at risk).
Secondary outcome: work performance – such as, presenteeism, reduced or impaired work capacity, quality of work or workability.	Where subpopulations of employees with common mental health problems were not taken as subpopulation in the data analysis.
Employees having one or more common mental disorders, or employees having symptoms of mental health problems, who ‘struggle at work’, assessed with self-assessment tools.	All severe mental disorders and personality disorders.
If burnout score is based on the Maslach burnout inventory: only if they score on emotional exhaustion as outcome for work performance.	Study on sickness absence, and thus reporting on employees on sick leave rather than still at work.
Individuals aged 18–65 years.	Economic impact studies.
Geographical/economic scope at first: globally.	
Study design a primary research study and published in peer-reviewed journals, reporting randomized controlled trials, cohort, case-control or cross-sectional studies, or qualitative descriptive (case) studies.	
Published in English, from 1995 and onwards.	

### Extraction and analysis process

For each study, the research team drafted one or more context-mechanisms-outcome (CMO) configurations, first independently and later discussed dually. These configurations described the causal links between context, mechanisms and outcomes (ie, SAW or WP). From each study, information from the methods, results and discussion section regarding relevant contextual factors or mechanisms leading to the selected outcomes were retrieved. Studies of high quality (see table 2) were used to form CMO configurations. Studies with insufficient methodological quality (answering ‘no’ to screening questions) were excluded and studies with risk of bias, rated as ‘medium quality’, were only used to support CMO configurations derived from high quality studies. Several iterative steps were followed to explore patterns within the extracted CMO configurations to develop middle range program theories, using ‘if…(context), then…(outcome), because of…(mechanisms)’ statements. Middle-range program theories are based on at least two included studies. In the final stage of the synthesis, we developed an explanatory framework, using the initial program theory to demonstrate what works, for whom, under what circumstances and how to promote SAW and WP.

**Table 2:** Overview of the characteristics and design of the studies. [CMD=common mental disorder; MMAT=mixed methods appraisal tool; LCA=latent class analysis; Obs=observational; RCT=randomized controlled trial; Int=intervention; SAW=stay at work: WP=work performance]

Author and reference	Type of study and study methodology	Number of participants	Study population (type of employees/sector)	Industry/ type of employees	MMAT score (H=rated as ‘high quality’; M=rated as ‘medium’)
**Articles reporting on both SAW and WP**					
Arends et al 2019 (51)	Obs: LCA	158	Dutch employees with CMD, mostly highly educated, who are in return to work trajectories	Various sectors	M: 3/5: no data on representativeness, low N for LCA
Birney et al 2016 (54)	Int: parallel two group RCT	300	US employees with depression, mostly middle-aged, Caucasian, female, highly educated	Unknown, part-time, fulltime and self employed	H: 4/5 blocked on race/ethnicity
Chen et al 2011 (69)	Obs: analytical cross-sectional study	452 (controls) 226 (cases)	Taiwanese young workers with depressive disorder at psychiatric clinics	Micro electronics engineers	H: 5/5
Daley et al 2009 (60)	Obs: cross sectional descriptive	308 patients	Canadian patients with symptoms of insomnia and 147 with insomnia syndrome, of whom 76.4% worked day shifts	Unknown	H: 5/5
Danielsson et al 2017 (6)	Obs: qualitative	27	Swedish workers, of various ages and job types, suffering from common mental disorders	Various sectors	H: 5/5
Duijts et al 2008 (45)	Int: RCT	57 (int) 61(control)	Dutch employees in 3 companies, with psychosocial health complaints, who are still working in health and educational sector at risk of sickness absence	Health Education	H: 4/5 low adherence to intervention (49%)
Dunner et al 2001 (63)	Int: before after studies	816	US patients with recurrent major depression who worked part-time or fulltime	Unknown	H: 5/5
Ebert et al 2016 (53)	Int: RCT	63	German employees with elevated stress levels, various sectors, mostly women and medium or high educated	Economy, service, social, IT, health, other	H: 5/5
Evans-Lacko & Knapp 2018 (29)	Obs: cross sectional survey	2985	Employees with self-reported depression from 15 different countries worldwide, mostly in Asian countries, from several sectors except marketing sector	Unknown, company size and working status varied	H: 4/5 Low response rate, representability of target population unclear
Hilton et al 2008 (41)	Obs: cross sectional study	60,556	Employees in New Zealand and Australia working in large companies, high level of psychological distress	Large public and private sector employers	H: 4/5 low response rate, blue collar underrepresented
Jha et al 2016 (81)	Int longitudinal study	331	US employed patients with nonpsychotic chronic or recurrent depression with current episode of more than 2 months	Unknown	M: 3/5 missing information about int., adherence and drop out
Johnson et al 2015 (64)	Int: controlled trial, not randomized.	40 of whom 20 in int. group	US working health care professionals, aged 18-65 years, who are at least 50% or higher employee status. With major depressive disorder, single episode or recurrent	Health care	H: 4/5: No sub group analysis or confounders due to small group of participants
Lerner et al 2010 (39)	Obs: longitudinal cohort study	286	US employees with depression, despite occupational group, married, gender, recruited through primary health care centres	Various sectors	H: 4/5: incomplete outcome data
Lerner at al 2020 (70)	Int: RCT	253	US veterans, with mild to moderate depression	Veterans	H: 5/5
Plaisier et al 2010 (59)	Obs, descriptive longitudinal	1035	Dutch workers with common mental health disorders	Unknown	H: 5/5
Plaisier et al 2012 (33)	Obs: cross sectional, descriptive	1522	Dutch workers who have an employer or who are self-employed (5%) with depression or anxiety disorder	Manual and non-manual jobs, self employed	H: 5/5
Richmond et al 2017 (36)	Int: prospective, quasi experimental design	344	US employees, mostly female (71%), white (87%) and non-Hispanic (81%), average education was 16 years, working for the government, with depression or anxiety	Diverse in human service providers	H: 4/5 incomplete outcome data
Ridge et al 2019 (48)	Obs: Qualitative	73	73 Australian and UK participants self-identified as having experienced depression	Professional or manual work	H: 4/5 quotes are rather general
Rost et al 2004 (47)	Int: RCT	198	US employed patients with major depression, mostly female (84.4), high school educated (88.5%), mostly full time employed (80%)	Administrators, managers, sales people, services	H: 4/5 missing information on intended treatment and utilization
Sahlin et al 2014 (50)	Int: before and after study	33	Swedish female health care workers suffering from high level of stress	Health care workers	H: Mixed method: 5/5 qual, 3/5 for quant: confounders not taken into account in analysis, not representative
Swanson et al 2011 (62)	Obs: cross sectional survey	367	US workers with any sleep disorder, with shift work	White, grey, blue collar and shift workers	M: 3/5: low response rate, no validated questionnaire
Telle et al 2016 (67)	Int: RCT one factorial design with two groups	99	German employees who subjectively felt mentally distress due to work-related issues, voluntary participation	13 different private corporations and federal and public organizations	M: 3/5: incomplete outcome data and low adherence to intervention
Uribe et al 2017 (57)	Obs: cross sectional	107	Colombian employees with major depression or double depression (N=107)	Unknown, employees part time, full time, self-employed	H: 5/5
van den Berg et al 2017 (40)	Obs: Cross sectional analytical	661	Dutch health care employees, mostly female and intermediate or high education, with a mental disorder	Health care workers	H: 5/5
van Mill et al 2013 (44)	Obs: epidemiologic cohort study	707 CMD and 728 without	Dutch depressed or anxious individuals who work 8 hours or more	Unknown	H: 5/5
Wang et al 2007 (55)	Int: RCT	604 of whom 304 in int. group	US employees with at least moderate depression, enrolled in a large managed behavioural health care company (insurance)	Diverse sectors: airline, insurance, banking, public utility, government, manufacturing	H: 5/5
Woo et al 2011 (49)	Int: controlled trial	106 and 91 healthy controls	South Korean employees with major depressive disorder	Employees in highly industrialized areas	H: 4/5 incomplete outcome data
**Articles reporting on SAW**					
Chakraborty & Subramanya 2013 (31)	Comparison Obs	43	Indian, industrial employees who work in an urban aeronautical industry who experience stress	Urban industrial employees	M: 3/5 selection bias
Cocker et al 2011 (56)	Obs: descriptive survey data	320	Australians with life time depression	Various sectors	H: 5/5
Corbiere et al 2016 (28)	Obs: qualitative	22	Canadian, mostly highly educated employees with symptoms of depression	Public, private and non-profit sector	H: 4/5 Recall bias, currently not working but during last 5 years
Hammond et al 2017 (30)	Obs: qualitative	6	Clinical psychologists in Australia who run a solo private practice, who experienced burnout maximum 2 years ago	Health care: psychologists	H: 5/5
Kawakami et al 1999 (65)	Int: RCT	81 in int, 77 in control group	Workers, mostly male, who are distressed and employed in Japan	Manufacturing company	M: 2/5: no information on randomization, no baseline comparison between groups, adherence unknown
Keus van de Poll et al 2020 (43)	Int: RCT	100	Swedish, mostly government workers using occupational health services suffering from CMD or work stress	Mainly public service employees	H: 4/5 not representative study population
Kok et al 2017 (32)	Obs: before and after study	1222	Dutch employees with an affective disorder	Unknown	H: 5/5
Laitinen-Krispijn & Bijl 2000 (34)	Obs: longitudinal study, follow up 1 year	3695	Dutch male employees with major depressive disorder, dysthymia, simple phobia and substance abuse/dependence	Unknown	M: 3/5: unclear outcome measure on duration of sick leave, few confounders
Leijten et al 2013 (37)	Obs: longitudinal study	354	Older Dutch employees with psychological problems (not specified)	Unknown	H: 5/5
Lexis et al 2009 (58)	Obs: prospective cohort	3339	Dutch employees with depressive complaints, from various organizations and companies	Various sectors	H: 5/5
Lexis et al 2011 (68)	Int: RCT	139	Dutch employees with depressive complaints, from various organizations and companies	Office workers	H: 5/5
Linden et al 2011 (52)	Int: before after study	44 outpatients	German employees, with generalized anxiety disorder in outpatient departments	Unknown	M: 3/5 missing info on representativeness and confounders
Mackenzie et al 2014 (46)	Int: RCT	93	Australian workers with depression, generalizes anxiety disorder and social phobia	Unknown	M: 2/5: randomization not explained, loss to follow up
Noordik et al 2011 (23)	Qualitative	14	10 Dutch women and 4 men, aged 25–58 (mean age 38) years, partially returned to work	Various sectors incl. health care	H: 5/5
O’Haire & Rodriguez 2018 (66)	Int: non RCT	141 in int., 75 control	US veterans working elsewhere and who were identified with PTSD after 9/11	Veterans	H: 4/5: 24,7% of population is working
Sado et al 2014 (61)	Obs: retrospective cohort	194	Japanese workers in a manufacturing company with repeated sick leave because of mental disorders	Manufacturing company	H: 5/5
Virtanen et al 2007 (38)	Obs: prospective study,	6663 female, 1323 male	Finnish local government employees and health care employees in public services with psychological distress	Public sector employees	H: 5/5
Vlasveld et al 2013 (101)	Obs: cross sectional	1425	Dutch workers with psychopathology (anxiety or depressive disorder)	Unknown	H: 5/5
Woodall et al 2017 (35)	Qualitative: semi-structured interviews	15	UK current or former service users with mental health conditions	Unknown	H: 5/5
**Articles reporting on WP**					
Adler et al 2006 (72)	Obs: longitudinal	286	US patients with major depressive disorder (N=105) or dysthymia (N=72) or both (N=109)	Mostly women 1) managers, professionals, and technicians; 2) sales, service, and support;	H: 5/5
Beck et al 2014 (78)	Obs prospective cohort	432	US working patients, on routine depression treatment	Unknown	H: 4/5: work context not in analysis
Bertilsson et al 2013 (74)	Obs qualitative	17	Swedish persons with CMD employed in regular job market, mainly women	Private and public sector	H: 4/5 late reflection on data
Danielsson et al 2020 (84)	Int: pilot RCT	147	Swedish employees with CMD, mainly female, on work-directed rehabilitation	Various sectors	H: 5/5
Furukawa et al 2012 (75)	Int: RCT non-blinded	108, of whom 58 in int. group	Japanese currently employed, mostly male, fulltime workers with minor depression at a large manufacturing company	Manufacturing company	H: 5/5
Haslam et al 2005 (71)	Obs: Qualitative	74	UK workers with personal experience of anxiety/depression in the previous 2 years and who are mostly (2/3) uncompliant with medication	Various sectors	H: 4/5 the interpretation of results insufficiently supported by data
Kim et al 2019 (73)	Obs: cross sectional	173	South Korean workers with depression	Various sectors	H: 5/5
Lam et al 2011 (80)	Int: pilot study	31	Canadian health agency workers (predominantly women, above 40), with symptoms of depression, counselling is purchased by employer and self-referred to the EAP	Health care	M: 4/5: small pilot study, self-referred to intervention, no confounders in analysis
Lappalainen et al 2013 (77)	Int: small scale RCT	11 int and 12 in control (waiting list)	Finnish male workers with stress and mood problems	Unknown	M: 2/5: no information on randomization, self-assessed outcome, no blinding
Lindsater et al 2018 (76)	Int: RCT	50 int. 50 in control	Swedish employees (of whom 82% employed full time or part-time), with adjustment disorder or exhaustion disorder	National sample	H: 5/5
Loukine et al 2016 (42)	Obs: cross-sectional	2528	Canadian workers with self-reported mood or anxiety disorders	Unknown	H: 5/5
Nigatu et al 2015 (79)	Obs: descriptive longitudinal	555	Dutch employees, currently having a major depression or anxiety disorder, mostly white collar workers	Unknown	H: 5/5
Okajima et al 2020 (83)	Int: RCT	92	Young Japanese employees with insomnia	Mostly office employees	H: 4/5: many lost to follow up
Petersson et al 2018 (82)	Int: RCT	132	Swedish Patients with mild to moderate depressive disorder	Various sectors, white- / blue collar	M: 3/5: low adherence and incomplete outcome data
Rothermund et al 2016 (102)	Int: controlled obs. trial	367	German employed patients of whom N=174 use psychotherapeutic consultation in the workplace	Three companies, unknown	H: 5/5

## Results

**Figure 1:**
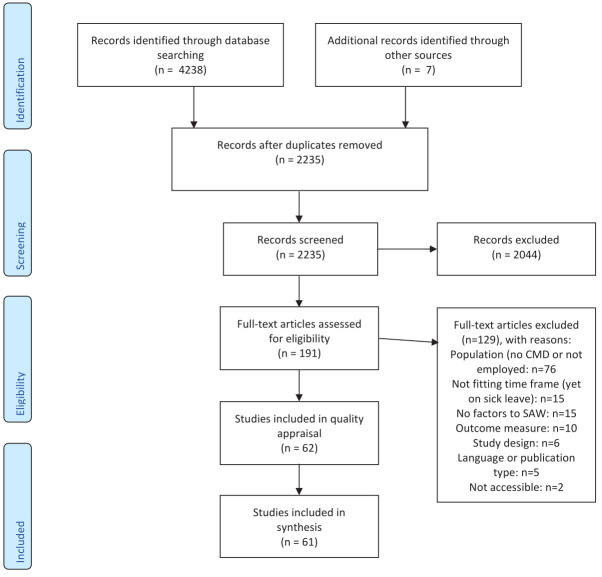
PRISMA flow chart of study inclusion process.

The search process yielded 2235 records, shown in figure 1. Screening on title and abstract led to the exclusion of 2044 articles, resulting into 191 articles for full text screening. After full text screening and quality appraisal, 61 articles were included. One study was excluded due to insufficient methodological quality. Studies ranked as medium quality were characterized by relatively low response rates or incomplete outcome data, or missing information regarding adherence and randomization procedures. The majority of the studies used quantitative data (N=53), only seven studies used qualitative data and one study used mixed methods. Table 2 provides an overview of characteristics of the included studies per outcome. Below, we first present the middle range program theories, which frame mechanisms and contextual factors that facilitate SAW, followed by the middle range program theories that facilitate WP.

**Figure 2:**
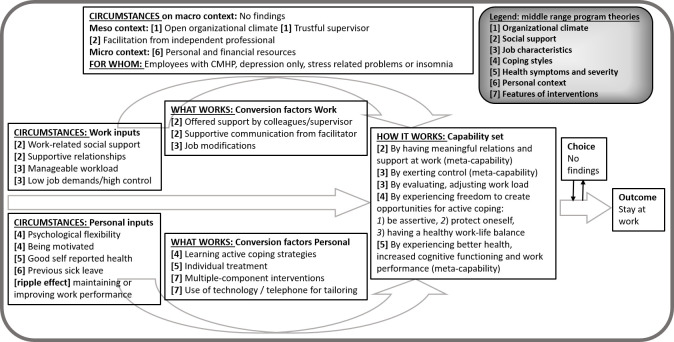
How to promote staying at work (SAW) among employees with CMHP, framed by Capability-for-Work model, based on 45 studies.

**Figure 3:**
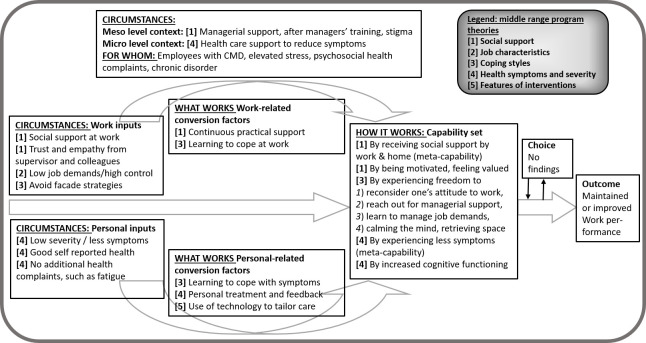
How to promote work performance: framed by Capability-for-Work model, based on 39 studies

Tables 3 and 4 present the summary of mechanisms that facilitate each outcome, SAW and WP, respectively. To explain the causal relations between context, mechanisms and outcomes we describe each middle range program theory. Thereafter, we present our findings in an explanatory framework. Figures 2 and 3 depict *what works, for whom, under what circumstances and how*, refining the Capability-for-work model. In-depth information regarding the data synthesis of CMO configurations per study, leading to the middle range program theories, is presented in supplementary Appendix B.

**Table 3:** Mechanisms that facilitate stay at work (SAW), among employees with CMHP.

Theme of program theory	Mechanisms regarding SAW
Organizational climate	Open organizational climate
	Trustful and available supervisor
	Openness from supervisor
	Employee mirrors supervisor
Social support	Offered adequate and timely support
	Supportive relationships with colleagues and supervisor
	Meaningful relations at work
	Work-related social support: being heard about work-related problems
	Facilitator from independent professional
	Supportive communication from facilitator: an encouraging attitude and knowhow about employment issues and workplace
Perceived job characteristics	Manageable workload
	Low job demands/high job control through exerting control over own work
	Job modifications and making adjustments at work
	Absence of overtime/over hours and high job strain
Coping styles	Psychological flexibility
	Being highly motivated for work
	Talk about symptoms
	Learning active coping skills, exerting control over own work, gaining mastery of symptoms, adjusting and evaluating workload
Health symptoms and severity	Good self-reported health
	No additional health complaints
	Individual treatment: pharmaco-/psychotherapy, stress reduction programs
	Better work performance (productivity)
	Decreased exhaustion
	Increased cognitive functioning
Personal context	Previous sick leave due to CMHP
	Personal resources (being married
	Financial resources (owning a house, being self-employed)
Features of SAW interventions	Multiple components
	Use of online or telephone support in addition to face to face care
	Tailoring care, to transfer skills into daily life

**Table 4:** Mechanisms that facilitate work performance (WP).

Theme of program theory	Mechanisms WP (outcome 2)
Social support	Managerial support, after training
	Trust and empathy received by employee
	Continuous practical job support from colleagues or supervisor
	Social support at work and at home or from clinician
Perceived job characteristics	Perceived low demands and high control
Coping styles	Avoid façade, to compensate shortcomings is counterproductive
	Learning to manage job
	Reach out for supervisor support
	Reconsider ones attitude to work
	Calming mind and retrieve space
	Learning to cope with symptoms
Health symptoms and severity	Good self-reported health, Lower severity / less symptoms
	Absence of chronicity or additional health complaints
	Individual treatment: psychotherapy, pharmacotherapy
	Increased cognitive functioning
Features of WP interventions	Use of technology
	Tailoring care, to transfer skills into daily life

### Stay at work (SAW)

The mechanisms, presented in table 3, reveal how organizational climate, social support in the work context, and perceived job characteristics enable employees with CMHP to stay at work. Furthermore, coping, severity of mental health symptoms, the personal context and features of interventions are factors affecting the chance for SAW.

*Middle-range program theory 1: Organizational climate*. Trustful relationships in which the supervisor shows openness to talk about mental health conditions in an open climate in general, may contribute to SAW among employees with depression because (a lack of) openness by supervisors is mirrored by employees (28, 29).

*Middle-range program theory 2: Social support*. Adequate and timely social support and supportive relationships, from colleagues but particularly supervisors who are willing to assist and listen to work related problems, increase the chance for SAW among employees with CMHP because this helps to obtain a manageable workload (23, 30–34). Facilitation, by either a mental health professional or job retention specialist – who (i) acts independently, with sympathy and pragmatism (ii) provides an expert insight and (iii) is familiar with the workplace – also improves the likelihood to stay at work (23, 35, 36).

*Middle-range program theory 3: Perceived job characteristics*. There is an inconsistent pattern with regard to job demands and control and its effect on SAW. A possible program theory, based on CMO configurations, could be that experiencing low job demands and high job control helps an employee to exert control over one’s own work, including adjustments that can be made (32, 33, 35, 37–40). Heavy workload, overtime and high job strain reduce the chance for SAW, among employees with stress or depression (28, 30, 31, 38). Job modifications help, however in a different way for white- versus blue-collar workers, due to the type of duties and work context (23, 30, 41–43).

*Middle-range program theory 4: Coping styles*. A lack of adaptive skills, due to reduced psychological flexibility and a different perspective on situations, reduces the capacity to bear responsibilities, which in turn has a negative effect on SAW (30, 32, 44). Useful coping skills for SAW are: being more alert on signals of reduced mental health, reading and understanding own signals, exerting control over one’s own work and workload, balancing positive and negative influences of work, making adjustments and informing colleagues, protecting oneself, taking control, and being assertive (23, 28, 30, 35, 37, 45–49). Also, being highly motivated towards the job increases the likelihood for SAW (23, 31, 50, 51). Adversely, employees who do not talk about their depression, hide themselves or deny their symptoms have a higher risk of absenteeism (6, 28–30, 34, 48). Improving active coping skills and advancing self-management in daily life subsequently contribute to SAW (36, 46, 50, 52–54) by addressing work in counselling besides personal problems (36, 43, 55)

*Middle-range program theory 5: Health symptoms and severity*. Better mental and physical health contributes to SAW because the employee’s experience of lower severity of symptoms leads to improvement in WP (by increased cognitive functioning or decreased exhaustion) (30, 33, 39, 56–60). Likewise, facing additional health complaints as well as previous sick leave, decreases the chance for SAW (44, 56, 60–62). Interventions offering psychotherapy or pharmacotherapy seem more effective than preventative treatment or stress reduction interventions (45, 47, 50, 52, 54, 62–68).

*Middle-range program theory 6: Personal context*. Personal characteristics may contribute to SAW based on possible underlying mechanisms, such as financial drive by owning a house, being self-employed, or being married (30, 33, 38, 56). Employees with depressive disorders who had more life events, personal problems or exposures in other life domains than work may experience tension or confusion about symptoms, leading to more absence days (28, 30, 38, 69).

*Middle-range program theory 7: Features of interventions*. If interventions focus on multiple components, for example if they target both personal inputs (symptom reduction and coping with symptoms) and work inputs (coping at the workplace or a better work-related health), this may lead to an increased likelihood for SAW (35, 46, 47, 49, 50, 52–54). In these interventions, using online or telephone support systems in addition to face-to-face care is successful because it (i) increases adherence and better access to early and regular screening and (ii) tailors messages to needs and integrates learned skills into daily life (46, 47, 53–55). Preventative, worksite-based job retention interventions or adding a work-focused intervention to integrated care did not seem effective on the outcome of SAW (45, 67, 68, 70).

*Explanatory framework to stay at work based on the Capability-for-Work model*. Based on the initial program theory and the presented middle range program theories, figure 2 depicts an explanatory framework for SAW. The mechanisms (the ‘how’) are mainly to be found under conversion factors and the capability set. The circumstances that facilitate SAW are to be found under Context on macro, meso, micro level and Personal- and Work inputs. We suppose that employees with CMHP can realize SAW through the following set of capabilities: (i) having meaningful relations and social support at work, (ii) exerting control, (iii) evaluating and adjusting the workload, (iv) experiencing freedom to create opportunities for active coping, (v) and experiencing better health, increased cognitive functioning and work performance. Those capabilities reflect the employee “being able” as well as “being enabled” (15). We also found the so-called ‘ripple effects’, in which the outcome of one CMO configuration became the context or mechanisms for the next in the chain of causality. For example, interventions on symptomatology (mechanism) seem to reduce the severity of symptoms (outcome). This outcome acts as an input (severity of symptoms) on SAW (outcome).

### Work performance

Table 4 presents the summary of the mechanisms that facilitate WP (outcome 2) for employees with CMHP. Five middle-range program theories are proposed on how social support, perceived job characteristics, coping styles, health symptoms and severity and features of interventions promote the employee’s WP respectively.

*Middle-range program theory 1: Social support*. A work environment where supervisors feel comfortable to offer help and support to employees, helps employees to feel motivated and valued, which in turn may have a positive effect on their job performance (29, 71). Practical job support from colleagues and managerial support from supervisors, offered continuously while functioning at work despite CMHP, helps to improve WP as the employee experiences trust and empathy (6, 29, 33, 48, 51, 69, 71).

*Middle-range program theory 2: Perceived job characteristics*. There is inconclusive evidence on interventions regarding job characteristics and their beneficial effect on WP among employees struggling with CMHP. Some studies suggest the combination of (perceived) high job demands and low job control may reduce WP among employees with CMHP (39, 40, 72). However, other studies contradict this suggestion (33, 73).

*Middle-range program theory 3: Coping styles*. If employees with CMHP experience reduced capacity to work, they initially use working facade strategies (such as increasing hours or taking work home), compensating possible shortcomings to avoid reduced performance because of fear and perceived stigma from colleagues and supervisor. However, these strategies seem counterproductive, as they result in emotional exhaustion, dissatisfaction and loss of refueling in the long run (6, 41, 71, 74). Interventions (eg, counselling) prove to promote WP because they improve effective coping styles in the long term. Examples of these interventions are (i) reconsidering one’s attitude to work, (ii) reaching out for supervisor support, (iii) learning new approaches to manage job demands, and (iv) calming the mind and retrieving space for recovery (6, 36, 39, 53, 54, 67, 75–77).

*Middle-range program theory 4: Health symptoms and severity*. Self-rated health and severity of symptoms are important predictors of WP among employees with depression, anxiety or sleep disorder because once the employee experiences less symptoms, work productivity improves (39, 57, 59, 62, 72, 78). Chronicity of symptoms has shown to reduce WP (33, 51, 59, 79). Interventions to reduce symptoms result in increased cognitive functioning, a pro-active attitude towards change, better mental-interpersonal task performance, improved time management and output, and subsequently to increased WP (36, 47, 49, 50, 53, 55, 64, 67, 76–78, 80, 81). Among employees with stress, interventions improve stress recovery and symptom management, which subsequently leads to improved productivity (50, 53, 64).

*Middle-range program theory 5: Features of intervention*. Interventions that use technology, through email, phone or app, may reduce mental health symptoms as well as work limitations. The use of these technologies helps to better monitor the employee’s behavior by tailoring the interventions with personal feedback, fostering belief changes and facilitating the transfer of training components to daily life (53, 55, 70, 76, 77, 80, 82–84)

*Explanatory framework on work performance based on the Capability-for-Work model*. An explanatory framework on how to realize WP among employees with CMHP is presented in figure 3. This figure illustrates that both personal- and work-related conversion factors promote WP through a set of capabilities. The capability set consists of (i) receiving social support from work and home, (ii) being motivated and feeling valued, (iii) experiencing freedom for active coping, and (iv) experiencing less symptoms and increased cognitive functioning. Where WP acts as a goal (outcome on its own), it also acts as a capability for SAW. This may support the idea of meta-capability suggested by Venkatapuram (85): being a capability in itself and also conditional (contextual factor) for achieving other capabilities. Capabilities may or may not result into work outcomes due to constrained or limited choices, as proposed in the Capability-for-Work model. Unfortunately, the included studies did not provide insights in the opportunity to make individual choices to achieve both work outcomes (see figure 2 and 3).

## Discussion

This paper provides a systematic realist review of studies that have assessed work participation among employees with CMHP. This review (i) contributes to the development of a more uniform definition of the concept SAW among the study population at risk of negative work outcomes due to CMHP, (ii) identifies mechanisms that promote work participation through the outcomes of SAW and WP (iii), sheds light on how the work context may promote work participation in practice and research, and (iv) provides an explanatory framework using middle range program theories, based on the Capability-for-Work model. These contributions, their implications for practice and future research as well as the limitations of the present study are discussed below.

### Contributions of the present study

The present study adds to our understanding of the complex, multifactorial process of work participation for employees with CMHP. The overall findings of this review are consistent with the findings of previous reviews on related outcomes, finding theoretical support for the dynamic interrelation between personal factors and work-related factors leading to work participation for employees with CMHP (5, 8, 86). However, our review also shows *how* social support in the work context, perceived job characteristics, coping styles and better experienced health may promote WP and SAW. Furthermore, insight is given in how organizational climate and personal context promote SAW and what features of interventions seem effective. In addition, the findings of this review shed light on underlying mechanisms towards an adequate, supportive, work environment that enables employees with CMHP to remain at work. Because we used a systematic realist review approach rather than summarizing factors that may not provide insights into causal relationships, we were able to “unpack” each mechanism, and reveal under what circumstances these mechanisms lead to the outcome of interest. In this way, it explains what often is experienced by practitioners in individual cases and is hard to support by empirical evidence due to averaged, usually small effects in quantitative studies.

Notwithstanding all efforts regarding preventative mental health interventions, our findings call for more attention to employees already facing CMHP in the work context, in line with the recommendations of the OECD and other researchers (5–7, 87). We operationalized SAW in such a way that it includes employees with CMHP who are currently working or reported partly sick. Interestingly, we observed in the review process that the current research agenda is still focused on absenteeism and return to work concerning employees with CMHP rather than SAW, despite the growing evidence base on prevention and positive psychology in the general working population (88). A possible explanation could be that the phase of being on sick leave or absent as a negative work outcome is directly related to costs of employers and society as a whole and thus of a greater interest in research and practice. Besides, being absent is more visible than being at work while being affected by CMHP. Signals of CMHP usually develop silently and slowly, making it harder for employers to signal and intervene. Also, CMHP are often stigmatized, making it hard for employees to decide whether or not to disclose their condition to their employer (89). This supports our decision to include both diagnosed and self-reported CMHP in this study. To gain insight in the promotion of work participation in a group of at-risk employees, we choose not to emphasize the highly discussed boundary between normality and pathology. Because complaints are often dynamic and fluctuating, such a clear distinction is not necessary for the purpose of this review. We found that the mechanisms and contexts to promote work participation apply to those employees with psychological symptoms in the subthreshold group, to those who did not seek help or had no access to care, as well as to employees with a diagnosed common mental disorder.

Regarding the retrieved mechanisms, more attention in the scientific literature was given to (intervening on) personal factors than work-related or organizational factors. This implies that, in interventions that promote work participation, efforts and effects seem to be attributed to the person rather than the work situation. This is not in line with the literature showing that work-related factors have great causal effects on sustainable work participation among the general working population. For example, the Job Demands Resource theory suggests that in order to effectively continue working despite facing CMHP, solutions can be found in the work context and job designs, more than intervening (only) on personal factors (90). Besides, despite our study approach to search in each included study for contextual factors, it was difficult to identify the organizational circumstances in which each mechanism or outcome occurred apart from the pre-defined intervention components. However, even if organizational circumstances were not analyzed explicitly, we succeeded to identify mechanisms that refer to the role of employers in supporting employees to stay at work (receiving supervisor support, being offered job modifications). This supports the evidence regarding the important responsibility of employers in facilitating employees with CMHP (13, 86, 91, 92). Therefore, more insight into work-related mechanisms and circumstances leading to SAW is needed to develop effective organizational interventions for employees with CMHP (93).

The use of the Capability-for-Work model contributed to the findings of our review in three ways. First, considering the plethora of CMO configurations derived from 61 studies, this model helped us to arrange factors and understand causal effects and underlying mechanisms. As such, we could distinguish inputs (pre-existing work- or personal factors that are often non-changeable) from conversion factors and capabilities (often changeable). Through a capability approach lens, mechanisms (how and why) were identified as conversion factors and capabilities. More specifically, we found that both personal conversion factors and social, work-related conversion factors are needed to realize capabilities to work (20). The framework adds to the understanding of causal relationships between all factors and the outcomes SAW and WP. Nevertheless, we emphasize that what may be a conversion factor or capability for one employee, can be a pre-existing personal factor for the other. Second, our review contributes to the development of the capability set for work, defined by Abma et al (94) in that we add to their seven capabilities, presenting specific capabilities for employees with CMHP. For example, the capability of building and maintaining meaningful contacts at work, is elaborated in our study by the capability of receiving work-related social support and having trustful relations with the supervisor and colleagues. Third, using the Capability-for-Work model, our review reveals that it is not the medical condition itself but its interactive effect with WP and circumstances that influence the employee’s functioning at work and ability to stay at work (95). Therefore, it will be more interesting to investigate whether employees are “being able” and “being enabled” to participate in work, and thus to unravel which set of capabilities is needed to do so, rather than solely to assess their medical condition. In this way, we highlight the importance of placing work participation in a wider spectrum of human development, shifting the focus from having a mental health condition as an impairing factor to the establishment of capabilities and choices (96).

### Implications for practice

This review provides valuable information to employers and occupational health professionals as to what implications they should focus on in order to promote work participation for employees with CMHP. The first practical implication refers to the importance of multilevel interventions from employers, addressing overarching themes on an organizational- and team-level combined with tailored interventions on the individual level. Employers could improve the work situation of employees with CMHP, and the teams and organizational culture they work in, by creating a socially safe, open working climate. On the individual level, employers could ask employees who are having a hard time at work what they can still do despite their problems, and what they need in their job or in the work context in order to remain at work. This way, the employer enables the employee to convert inputs and resources into capabilities. Employers should seek advice from occupational health professionals since they can support, on different levels and on both sides, the employer as well as the employee with CMHP.

Next, we highlight the need for early intervention, and suggest professionals to find ways to assess and intervene on capabilities and WP before employees report sick, besides assessing the employee’s (severity of the) condition or other pre-existing personal factors. Occupational health professionals can discuss individual short-term adjustments in the job or work context with the employee and employer. For long term solutions, those professionals can support employers to detect a mismatch between the employee’s capabilities and the work (context).

In line with addressing employee’s abilities rather than problems, we recommend two ways to increase employee’s experience of freedom, often referred to as autonomy in the literature. On the side of healthcare and the psychological treatment of individuals with CMHP, we recommend (mental) healthcare professionals to address work-related problems in the consultation and to transfer lessons learned, such as active coping, to the work context. Likewise, we urge employers to facilitate work and a work environment where lessons learned can be practiced by employees, by enhancing autonomy or facilitating temporary job modifications (97). This may have the twofold effect of increasing employee’s capabilities and employee engagement as well as contributing to mentally healthy workplaces (95).

Finally, providing continuous social and practical support at the workplace is crucial to promote work participation. Employers should take preventative measures whilst the employee is still at work, for example by educating supervisors and colleagues on reading signals and talking about mental health. Also, employers can increase supervisors’ skills on ways to offer support to employees and increase know-how of situations that require referral to occupational health professionals.

### Recommendations for future research

The following recommendations for future research result from this study. First, our review showed that WP acts as a meta-capability for SAW, illustrating a possible parallel link between CMHP and the level of work performance during the phase of staying at work (98, 99). Further research is needed to test the link between both work outcomes, verifying whether and how WP can be used as a means to decrease the severity of CMHP, resulting in an increased chance to stay at work. Second, additional research is warranted to further develop the Capability-for-work model on work participation for employees with CMHP. We recommend the use of empirical data to test the newly presented set of capabilities among employees with CMHP in work participation. Besides, to further explore the causal relations presented in the explanatory framework, mean correlations that exist in the study population on group level could be tested but also underlying mechanisms that occur on an individual level. Third, we recommend realist evaluation as an approach to “unpack” underlying mechanisms and contextual factors in order to develop effective organizational interventions. As our research included only one mixed methods study and few qualitative studies, we cannot emphasize enough on the integration of process and outcome evaluation, using novel, mixed methods evaluation designs (12, 100).

In a next step, based on our review results, we will develop and evaluate a multilevel workplace intervention. This intervention aims to improve supervisor’s skills and competence to support employees with CMHP and create a work context that promotes work participation.

### Strengths and limitations

This systematic realist review provides a comprehensive overview of mechanisms and contextual factors promoting work participation. By using a realist approach, we succeeded to unravel mechanisms and their causal relationship with the work environment and selected outcomes. The realist data extraction- and data analysis process was time-consuming. However, it seemed valuable as the rigorous understanding of not only what works, but also under what circumstances and how work participation occurs, resulted in more practical contributions. Furthermore, we stimulate the debate among researchers on the understanding of work participation by contributing to theory development of the Capability-for-work model regarding various work participation outcomes.

The present review has a number of limitations that must be addressed. First, it could be argued that the heterogeneity in the type of studies and measures of outcomes led to CMO configurations with different levels of relevance or rigor. To overcome this, two researchers conducted each review step independently, using clearly defined concepts, inclusion and exclusion criteria and assessment tools. Also, the researchers discussed every defined CMO configuration. A second limitation refers to the dichotomous outcome of SAW. Due to the inconsistent definition of SAW in the literature, we screened a plethora of studies using the opposite outcome of SAW, reported as absenteeism or sickness absence. Barriers leading to absenteeism are not automatically facilitators of SAW, so the outcome of absenteeism is not irreversible as such. Therefore we only included studies that compared employees with CMHP who were absent to similar employees who stayed at work. A third limitation is that although we used information regarding context, mechanisms or implementation from the discussion section in publications, the contextual information was only explicitly provided to a certain extent (study population, employment sector). Where information regarding the context of the study was not given, we cannot know under what circumstances certain interventions work. This is a common limitation of realist synthesis and, therefore, is also relevant to our study. For an in-depth discussion on the use of realist research, we refer to our protocol paper (21).

### Concluding remarks

This systematic realist review revealed mechanisms and contextual factors that promote both WP and SAW for employees with CMHP. In these situations, the work environment can support employees to participate at work. Program theories using a realist approach reveal how the organizational climate, social support in the work context, and perceived job characteristics enable employees to participate at work. Furthermore, coping styles, severity of mental health symptoms, the personal context and features of interventions enable employees to participate at work. By providing an overview of recent scientific literature, this study provides valuable insights and practical implications for employers, occupational health professionals and researchers in the development and evaluation of evidence-based interventions. Novel explanatory frameworks, based on the Capability-for-Work model, present causal relations between personal- and work factors and a set of capabilities leading to SAW and WP. Finally, the study adds to the debate on using novel methodological research approaches such as realist synthesis, answering what works, for whom, under what circumstances and how.

## Supplementary material

Supplementary material

## References

[1] Thisted CN, Nielsen CV, Bjerrum M (2018). Work Participation Among Employees with Common Mental Disorders:A Meta-synthesis. J Occup Rehabil.

[2] OECD (2015). Organization for Economic Co-operation and Development. Fit Mind, Fit Job. From Evidence to Practice in Mental Health and Work.

[3] Andersen MF, Nielsen KM, Brinkmann S (2012). Meta-synthesis of qualitative research on return to work among employees with common mental disorders. Scand J Work Environ Health.

[4] Harvey SB, Modini M, Joyce S, Milligan-Saville JS, Tan L, Mykletun A (2017). Can work make you mentally ill?A systematic meta-review of work-related risk factors for common mental health problems. Occup Environ Med.

[5] Pomaki G, Franche RL, Murray E, Khushrushahi N, Lampinen TM (2012). Workplace-based work disability prevention interventions for workers with common mental health conditions:a review of the literature. J Occup Rehabil.

[6] Danielsson L, Elf M, Hensing G (2019). Strategies to keep working among workers with common mental disorders - a grounded theory study. Disabil Rehabil.

[7] OECD (2012). Sick on the Job?:Myths and Realisities about Mental Health and Work.

[8] Lagerveld SE, Bültmann U, Franche RL, van Dijk FJ, Vlasveld MC, van der Feltz-Cornelis CM (2010). Factors associated with work participation and work functioning in depressed workers:a systematic review. J Occup Rehabil.

[9] Rugulies R, Aust B (2019). Work and mental health:what do we know and how can we intervene?. Scand J Work Environ Health.

[10] Nexø MA, Kristensen JV, Grønvad MT, Kristiansen J, Poulsen OM (2018). Content and quality of workplace guidelines developed to prevent mental health problems:results from a systematic review. Scand J Work Environ Health.

[11] Muñoz-Murillo A, Esteban E, Ávila CC, Fheodoroff K, Haro JM, Leonardi M (2018). Furthering the Evidence of the Effectiveness of Employment Strategies for People with Mental Disorders in Europe:A Systematic Review. Int J Environ Res Public Health.

[12] Nielsen K (2017). Organizational occupational health interventions:what works for whom in which circumstances?. Occup Med (Lond).

[13] Higgins A, O'Halloran P, Porter S (2012). Management of long term sickness absence:a systematic realist review. J Occup Rehabil.

[14] Pawson R, Greenhalgh T, Harvey G, Walshe K (2005). Realist review--a new method of systematic review designed for complex policy interventions. J Health Serv Res Policy.

[15] van der Klink JJ, Bültmann U, Burdorf A, Schaufeli WB, Zijlstra FR, Abma FI (2016). Sustainable employability--definition, conceptualization, and implications:A perspective based on the capability approach. Scand J Work Environ Health.

[16] Sen AK, Nussbaum M, Sen A.K (1993). The quality of life. Capability and well-being.

[17] Weidel T (2018). Moving Towards a Capability for Meaningful Labor. J Human Dev Capabil.

[18] Bernardi A (2019). Using the Capability Approach and Organizational Climate to study Occupational health and safety. Insights into regional development.

[19] Nussbaum MC (2011). Creating Capabilities:The Human Development Approach.

[20] Bonvin JM (2012). Individual working lives and collective action. An introduction to capability for work and capability for voice. Transfer.

[21] van Hees SGM, Carlier BE, Blonk RW, Oomens S (2021). Understanding work participation among employees with common mental disorders:what works, for whom, under what circumstances and how?A systematic realist review protocol. Work.

[22] de Vries HJ, Reneman MF, Groothoff JW, Geertzen JH, Brouwer S (2012). Factors promoting staying at work in people with chronic nonspecific musculoskeletal pain:a systematic review. Disabil Rehabil.

[23] Noordik E, Nieuwenhuijsen K, Varekamp I, van der Klink JJ, van Dijk FJ (2011). Exploring the return-to-work process for workers partially returned to work and partially on long-term sick leave due to common mental disorders:a qualitative study. Disabil Rehabil.

[24] Nielsen K, Nielsen MB, Ogbonnaya C, Kansala M, Saari E, Isaksson K (2017). Workplace resources to improve both employee well-being and performance:A systematic review and meta-analysis. Work Stress.

[25] Wong G, Greenhalgh T, Westhorp G, Buckingham J, Pawson R (2013). RAMESES publication standards:realist syntheses. BMC Med.

[26] Moher D, Liberati A, Tetzlaff J, Altman DG, PRISMA Group (2009). Preferred reporting items for systematic reviews and meta-analyses:the PRISMA statement. PLoS Med.

[27] Pluye P, Gagnon MP, Griffiths F, Johnson-Lafleur J (2009). A scoring system for appraising mixed methods research, and concomitantly appraising qualitative, quantitative and mixed methods primary studies in Mixed Studies Reviews. Int J Nurs Stud.

[28] Corbière M, Samson E, Negrini A, St-Arnaud L, Durand MJ, Coutu MF (2016). Factors perceived by employees regarding their sick leave due to depression. Disabil Rehabil.

[29] Evans-Lacko S, Knapp M (2018). Is manager support related to workplace productivity for people with depression:a secondary analysis of a cross-sectional survey from 15 countries. BMJ Open.

[30] Hammond TE, Crowther A, Drummond S (2018). A Thematic Inquiry into the Burnout Experience of Australian Solo-Practicing Clinical Psychologists. Front Psychol.

[31] Chakraborty S, Subramanya AH (2013). Socio-demographic and clinical predictors of absenteeism - A cross-sectional study of urban industrial employees. Ind Psychiatry J.

[32] Kok AA, Plaisier I, Smit JH, Penninx BW (2017). The impact of conscientiousness, mastery, and work circumstances on subsequent absenteeism in employees with and without affective disorders. BMC Psychol.

[33] Plaisier I, de Graaf R, de Bruijn J, Smit J, van Dyck R, Beekman A (2012). Depressive and anxiety disorders on-the-job:the importance of job characteristics for good work functioning in persons with depressive and anxiety disorders. Psychiatry Res.

[34] Laitinen-Krispijn S, Bijl RV (2000). Mental disorders and employee sickness absence:the NEMESIS study. Netherlands Mental Health Survey and Incidence Study. Soc Psychiatry Psychiatr Epidemiol.

[35] Woodall J, Southby K, Trigwell J, Lendzionowski V, Rategh R (2017). Maintaining employment and improving health A qualitative exploration of a job retention programme for employees with mental health conditions. Int J Workplace Health Manag.

[36] Richmond MK, Pampel FC, Wood RC, Nunes AP (2017). The impact of employee assistance services on workplace outcomes:results of a prospective, quasi-experimental study. J Occup Health Psychol.

[37] Leijten FR, van den Heuvel SG, Ybema JF, Robroek SJ, Burdorf A (2013). Do work factors modify the association between chronic health problems and sickness absence among older employees?. Scand J Work Environ Health.

[38] Virtanen M, Vahtera J, Pentti J, Honkonen T, Elovainio M, Kivimäki M (2007). Job strain and psychologic distress influence on sickness absence among Finnish employees. Am J Prev Med.

[39] Lerner D, Adler DA, Rogers WH, Chang H, Lapitsky L, McLaughlin T (2010). Work performance of employees with depression:the impact of work stressors. Am J Health Promot.

[40] van den Berg S, Burdorf A, Robroek SJ (2017). Associations between common diseases and work ability and sick leave among health care workers. Int Arch Occup Environ Health.

[41] Hilton MF, Scuffham PA, Sheridan J, Cleary CM, Whiteford HA (2008). Mental ill-health and the differential effect of employee type on absenteeism and presenteeism. J Occup Environ Med.

[42] Loukine L, O'Donnell S, Goldner EM, McRae L, Allen H (2016). État de santé, limitations d'activité, restrictions professionnelles et degréd'invaliditéchez les Canadiens atteints d'un trouble de l'humeur ou d'anxiété. [Health status, activity limitations, work-related restrictions and level of disability among Canadians with mood and/or anxiety disorders]. Health Promot Chronic Dis Prev Can.

[43] Keus van de Poll M, Nybergh L, Lornudd C, Hagberg J, Bodin L, Kwak L (2020). Preventing sickness absence among employees with common mental disorders or stress-related symptoms at work:a cluster randomised controlled trial of a problem-solving-based intervention conducted by the Occupational Health Services. Occup Environ Med.

[44] van Mill JG, Vogelzangs N, Hoogendijk WJ, Penninx BW (2013). Sleep disturbances and reduced work functioning in depressive or anxiety disorders. Sleep Med.

[45] Duijts SF, Kant I, van den Brandt PA, Swaen GM (2008). Effectiveness of a preventive coaching intervention for employees at risk for sickness absence due to psychosocial health complaints:results of a randomized controlled trial. J Occup Environ Med.

[46] Mackenzie A, Harvey S, Mewton L, Andrews G (2014). Occupational impact of internet-delivered cognitive behavior therapy for depression and anxiety:reanalysis of data from five Australian randomised controlled trials. Med J Aust.

[47] Rost K, Smith JL, Dickinson M (2004). The effect of improving primary care depression management on employee absenteeism and productivity. A randomized trial. Med Care.

[48] Ridge D, Broom A, Kokanović R, Ziebland S, Hill N (2019). Depression at work, authenticity in question:Experiencing, concealing and revealing. Health.

[49] Woo JM, Kim W, Hwang TY, Frick KD, Choi BH, Seo YJ (2011). Impact of depression on work productivity and its improvement after outpatient treatment with antidepressants. Value Health.

[50] Sahlin E, Ahlborg G Jr, Matuszczyk JV, Grahn P (2014). Nature-based stress management course for individuals at risk of adverse health effects from work-related stress-effects on stress related symptoms, workability and sick leave. Int J Environ Res Public Health.

[51] Arends I, Almansa J, Stansfeld SA, Amick BC, van der Klink JJ, Bültmann U (2019). One-year trajectories of mental health and work outcomes post return to work in patients with common mental disorders. J Affect Disord.

[52] Linden M, Zubrägel D, Bär T (2011). Occupational functioning, sickness absence and medication utilization before and after cognitive-behavior therapy for generalized anxiety disorders. Clin Psychol Psychother.

[53] Ebert DD, Heber E, Berking M, Riper H, Cuijpers P, Funk B (2016). Self-guided internet-based and mobile-based stress management for employees:results of a randomised controlled trial. Occup Environ Med.

[54] Birney AJ, Gunn R, Russell JK, Ary DV (2016). MoodHacker Mobile Web App With Email for Adults to Self-Manage Mild-to-Moderate Depression:Randomized Controlled Trial. JMIR Mhealth Uhealth.

[55] Wang PS, Simon GE, Avorn J, Azocar F, Ludman EJ, McCulloch J (2007). Telephone screening, outreach, and care management for depressed workers and impact on clinical and work productivity outcomes:a randomized controlled trial. JAMA.

[56] Cocker F, Martin A, Scott J, Venn A, Otahal P, Sanderson K (2011). Factors associated with presenteeism among employed Australian adults reporting lifetime major depression with 12-month symptoms. J Affect Disord.

[57] Uribe JM, Pinto DM, Vecino-Ortiz AI, Gómez-Restrepo C, Rondón M (2017). Presenteeism, Absenteeism, and Lost Work Productivity among Depressive Patients from Five Cities of Colombia. Value Health Reg Issues.

[58] Lexis MA, Jansen NW, van Amelsvoort LG, van den Brandt PA, Kant I (2009). Depressive complaints as a predictor of sickness absence among the working population. J Occup Environ Med.

[59] Plaisier I, Beekman AT, de Graaf R, Smit JH, van Dyck R, Penninx BW (2010). Work functioning in persons with depressive and anxiety disorders:the role of specific psychopathological characteristics. J Affect Disord.

[60] Daley M, Morin CM, LeBlanc M, Grégoire JP, Savard J, Baillargeon L (2009). Insomnia and its relationship to health-care utilization, work absenteeism, productivity and accidents. Sleep Med.

[61] Sado M, Shirahase J, Yoshimura K, Miura Y, Yamamoto K, Tabuchi H (2014). Predictors of repeated sick leave in the workplace because of mental disorders. Neuropsychiatr Dis Treat.

[62] Swanson LM, Arnedt JT, Rosekind MR, Belenky G, Balkin TJ, Drake C (2011). Sleep disorders and work performance:findings from the 2008 National Sleep Foundation Sleep in America poll. J Sleep Res.

[63] Dunner DL, Kwong WJ, Houser TL, Richard NE, Donahue RM, Khan ZM (2001). Improved Health-Related Quality of Life and Reduced Productivity Loss After Treatment With Bupropion Sustained Release:A Study in Patients With Major Depression. Prim Care Companion J Clin Psychiatry.

[64] Johnson JR, Emmons HC, Rivard RL, Griffin KH, Dusek JA (2015). Resilience Training:A Pilot Study of a Mindfulness-Based Program with Depressed Healthcare Professionals. Explore (NY).

[65] Kawakami N, Haratani T, Iwata N, Imanaka Y, Murata K, Araki S (1999). Effects of mailed advice on stress reduction among employees in Japan:a randomized controlled trial. Ind Health.

[66] O'Haire ME, Rodriguez KE (2018). Preliminary efficacy of service dogs as a complementary treatment for posttraumatic stress disorder in military members and veterans. J Consult Clin Psychol.

[67] Telle NT, Moock J, Heuchert S, Schulte V, Rössler W, Kawohl W (2016). Job Maintenance through Supported Employment PLUS:A Randomized Controlled Trial. Front Public Health.

[68] Lexis MA, Jansen NW, Huibers MJ, van Amelsvoort LG, Berkouwer A, Tjin A Ton G (2011). Prevention of long-term sickness absence and major depression in high-risk employees:a randomised controlled trial. Occup Environ Med.

[69] Chen SW, Wang PC, Hsin PL, Oates A, Sun IW, Liu SI (2011). Job stress models, depressive disorders and work performance of engineers in microelectronics industry. Int Arch Occup Environ Health.

[70] Lerner D, Adler DA, Rogers WH, Ingram E, Oslin DW (2020). Effect of Adding a Work-Focused Intervention to Integrated Care for Depression in the Veterans Health Administration:A Randomized Clinical Trial. JAMA Netw Open.

[71] Haslam C, Atkinson S, Brown SS, Haslam RA (2005). Anxiety and depression in the workplace:effects on the individual and organisation (a focus group investigation). J Affect Disord.

[72] Adler DA, McLaughlin TJ, Rogers WH, Chang H, Lapitsky L, Lerner D (2006). Job performance deficits due to depression. Am J Psychiatry.

[73] Kim J, Kim YK, Leem SH, Won JU (2019). Association between job-related stress and experience of presenteeism among Korean workers stratified on the presence of depression. Ann Occup Environ Med.

[74] Bertilsson M, Petersson EL, Ostlund G, Waern M, Hensing G (2013). Capacity to work while depressed and anxious--a phenomenological study. Disabil Rehabil.

[75] Furukawa TA, Horikoshi M, Kawakami N, Kadota M, Sasaki M, Sekiya Y, GENKI Project (2012). Telephone cognitive-behavioral therapy for subthreshold depression and presenteeism in workplace:a randomized controlled trial. PLoS One.

[76] Lindsäter E, Axelsson E, Salomonsson S, Santoft F, Ejeby K, Ljótsson B (2018). Internet-Based Cognitive Behavioral Therapy for Chronic Stress:A Randomized Controlled Trial. Psychother Psychosom.

[77] Lappalainen P, Kaipainen K, Lappalainen R, Hoffrén H, Myllymäki T, Kinnunen ML (2013). Feasibility of a personal health technology-based psychological intervention for men with stress and mood problems:randomized controlled pilot trial. JMIR Res Protoc.

[78] Beck A, Crain LA, Solberg LI, Unützer J, Maciosek MV, Whitebird RR (2014). The effect of depression treatment on work productivity. Am J Manag Care.

[79] Nigatu YT, Reijneveld SA, Penninx BW, Schoevers RA, Bültmann U (2015). The longitudinal joint effect of obesity and major depression on work performance impairment. Am J Public Health.

[80] Lam RW, Lutz K, Preece M, Cayley PM, Bowen Walker A (2011). Telephone-administered cognitive-behavioral therapy for clients with depressive symptoms in an employee assistance program:a pilot study. Ann Clin Psychiatry.

[81] Jha MK, Minhajuddin A, Greer TL, Carmody T, Rush AJ, Trivedi MH (2016). Early Improvement in Work Productivity Predicts Future Clinical Course in Depressed Outpatients:Findings From the CO-MED Trial. Am J Psychiatry.

[82] Petersson EL, Wikberg C, Westman J, Ariai N, Nejati S, Björkelund C (2018). Effects on work ability, job strain and quality of life of monitoring depression using a self-assessment instrument in recurrent general practitioner consultations:A randomized controlled study. Work.

[83] Okajima I, Akitomi J, Kajiyama I, Ishii M, Murakami H, Yamaguchi M (2020). Effects of a Tailored Brief Behavioral Therapy Application on Insomnia Severity and Social Disabilities Among Workers With Insomnia in Japan:A Randomized Clinical Trial. JAMA Netw Open.

[84] Danielsson L, Waern M, Hensing G, Holmgren K (2020). Work-directed rehabilitation or physical activity to support work ability and mental health in common mental disorders:a pilot randomized controlled trial. Clin Rehabil.

[85] Venkatapuram S (2011). Health Justice:An argument from the Capabilities Approach.

[86] Harvey S, Joyce S, Tan L, Johnson A, Nguyen H, Modini M (2014). Developing a mentally healthy workplace:a review of the literature.

[87] Joyce S, Modini M, Christensen H, Mykletun A, Bryant R, Mitchell PB (2016). Workplace interventions for common mental disorders:a systematic meta-review. Psychol Med.

[88] Bolier L, Haverman M, Westerhof GJ, Riper H, Smit F, Bohlmeijer E (Feb). Positive psychology interventions:a meta-analysis of randomized controlled studies. BMC Public Health 2013.

[89] Brouwers EP, Joosen MC, van Zelst C, Van Weeghel J (2020). To Disclose or Not to Disclose:A Multi-stakeholder Focus Group Study on Mental Health Issues in the Work Environment. J Occup Rehabil.

[90] Bakker AB, Demerouti E (2017). Job demands-resources theory:taking stock and looking forward. J Occup Health Psychol.

[91] MacEachen E, Clarke J, Franche RL, Irvin E, Workplace-based Return to Work Literature Review Group (2006). Systematic review of the qualitative literature on return to work after injury. Scand J Work Environ Health.

[92] Joosen MC, Lugtenberg M, Arends I, van Gestel HJ, Schaapveld B, Terluin B (2021). Barriers and Facilitators for Return to Work from the Perspective of Workers with Common Mental Disorders with Short, Medium and Long-Term Sickness Absence:A Longitudinal Qualitative Study. J Occup Rehabil.

[93] Hazelzet E, Picco E, Houkes I, Bosma H, de Rijk A (2019). Effectiveness of Interventions to Promote Sustainable Employability:A Systematic Review. Int J Environ Res Public Health.

[94] Abma FI, Brouwer S, de Vries HJ, Arends I, Robroek SJ, Cuijpers MP (2016). The capability set for work:development and validation of a new questionnaire. Scand J Work Environ Health.

[95] Gray P, Senabe S, Naicker N, Kgalamono S, Yassi A, Spiegel JM (2019). Workplace-Based Organizational Interventions Promoting Mental Health and Happiness among Healthcare Workers:A Realist Review. Int J Environ Res Public Health.

[96] Trani JF, Bakhshi P, Bellanca N, Biggeri M, Marchetta F (2011). Disabilties through the Capability Approach lens:implications for public policies. Alter. European Journal of Disability Research.

[97] Zafar N, Rotenberg M, Rudnick A (2019). A systematic review of work accommodations for people with mental disorders. Work.

[98] Corbiere M, Negrini A, Dewa CS, Loisel P, Anema J.R (2013). Mental Health Problems and Mental Disorders:Linked Determinants to Work Participation and Work Functioning. Handbook of Work Disability:Prevention and Management.

[99] Brady GM, Truxillo DM, Cadiz DM, Rineer JR, Caughlin DE, Bodner T (2020). Opening the black box:examining the nomological network of work ability and its role in organizational research. J Appl Psychol.

[100] Boot CR, Bosma AR (2021). How qualitative studies can strengthen occupational health research. Scand J Work Environ Health.

[101] Vlasveld MC, van der Feltz-Cornelis CM, Anema JR, van Mechelen W, Beekman AT, van Marwijk HW (2013). The associations between personality characteristics and absenteeism:a cross-sectional study in workers with and without depressive and anxiety disorders. J Occup Rehabil.

[102] Rothermund E, Gündel H, Rottler E, Hölzer M, Mayer D, Rieger M (2016). Effectiveness of psychotherapeutic consultation in the workplace:a controlled observational trial. BMC Public Health.

