# Supplementary material for: Towards a better understanding of work participation among employees with common mental health problems: a systematic realist review
Source: Scand J Work Environ Health. 2022 Mar 31;48(3):173–89. doi: 10.5271/sjweh.4005 (PMC9523465; doi:10.5271/sjweh.4005)
Supplement: Supplementary material [file SJWEH-48-173-S001.pdf]

Towards a better understanding of work participation among employees with common mental health problems: a systematic realist review[1]

by Suzanne GM van Hees, MSc,[2] Bouwine E Carlier, PhD, Emma Vossen, PhD, Roland WB Blonk, PhD, Shirley Oomens, PhD

1. Supplementary material
2. Correspondence to: Suzanne van Hees, Occupation and health research group, HAN University of Applied Sciences, P.O. Box 6960, 6503 GL Nijmegen, The Netherlands. [E-mail: Suzanne.vanhees@han.nl]ORCID ID: 0000-0002-0281-7909

Appendix A.

Methods in detail.

Rationale for using realist synthesis

Our rationale for conducting a systematic realist literature review (SRR) is to gain insight in the mechanisms that promote work participation and in how the context may influence the effect of these mechanisms on the selected work outcomes, that is context-mechanism-outcome relations (1).

Definition of realist terms

Middle range program theories: theories that lie between the working hypotheses from the researchers who design and evaluate an intervention and the all-inclusive systematic efforts to develop a theoretical framework that may explain all of the observed uniformities of social behaviour, social organization and social change (2).
Context: context refers to "something that enables or disables the current mechanism of interest" (2). It often refers to the 'setting' of programs and research. As conditions change over time, the context may also reflect aspects of those changes while the program is implemented.
Mechanisms: mechanisms are underlying entities, processes or structures that lead to influence the outcome (3). This can refer to processes within the participant of an intervention or exposure (resources), their cognitive and emotional responses (reasonings), typically related to the intervention or exposure being offered. But it can also refer to processes within the context, like a company in which the participant is working.
Outcome: an outcome is what can be measured in terms of impact across the target population, using measurable or measured indicators. Outcomes can be considered as quantitative or qualitative, and intended or unintended (4).
CMO configuration: describes the causal links between context, mechanisms and outcome considered as causative explanations pertaining to the evidence on the topic of interest (2).
Ripple effect: the outcome of one CMO configuration becomes the context or mechanisms for the next in the chain of causality (5).

Procedure

The SRR followed the steps and procedures outlined by RAMESES publication Standards for Realist Synthesis (6). Details of the protocol for this SRR are registered on PROSPERO and can be accessed at https://www.crd.york.ac.uk/prospero/display_record.php?RecordID=108913. Regarding the search strategy and study selection, we adhered to the PRISMA guidelines for the conduct of systematic reviews (7).

Step 1. Developing an initial program theory	The Capability-for-Work model formed our initial program theory (8). The capabilities represent a person's opportunity and ability to achieve certain outcomes, taking into account someone's particular circumstances (9). It incorporates the following work capabilities: 1) the use of knowledge and skills, 2) the development of knowledge and skills, 3) involvement in important decisions, 4) building and maintaining meaningful contacts at work, 5) setting own goals, 6) having a good income and 7) contributing to something valuable (8). Derived from this model, we hypothesised that work participation is determined by the way an employee succeeds in converting personal- and work inputs and resources (i.e., conversion factors) into capabilities and subsequently into work functioning such as SAW and WP (8). Inputs are the personal resources (e.g. health, knowledge) or workplace resources (e.g. a set of tasks) and conversion factors refer to the process of converting one's inputs to tangible capabilities, resulting into work functioning that the employee chooses to achieve. These personal- and working factors interact with the context at the individual, organisational and societal level.
Step 2. Search strategy and literature search	All scientific peer-reviewed studies available between the 1st of January 1995 and the 26th of June 2020 were retrieved in this SRR. We conducted a computer-based search in the following databases, Pubmed, Medline, PsycInfo, Embase, Cochrane, Cinahl and Web of Science. The search string, developed in consultation with an independent health research librarian, comprised of three groups of keywords, 1) employees with common mental disorders or psychological complaints, 2) stay at work and 3) (reduced) work performance (Appendix A).
Step 3. Selection and appraisal of studies	Endnote was used to deduplicate references from the various databases. Thereafter, all references were imported into the software of Rayyan (10). Screening of references consisted of two steps. First, two independent researchers (SH and BC; SH and EV) dually assessed the studies' relevance with inclusion and exclusion selection criteria during the title and abstract screening, being blinded from each other's decision (refer to table 1). The level of agreement after title and abstract screening was 97% and 69 conflicts were discussed between each of the two research teams. This step led to a selection of 191 citations, from the 2,235 citations after removal of duplications. Next, two independent researchers (SH and BC; SH and EV) dually read the full texts and were blinded to decide whether articles should be included for data extraction. Researchers based their decision on the selection criteria as well as whether the findings contribute to theory testing of the initial program theory and its refinement and thus contain contexts, mechanisms and outcomes of interest. Conflicts in this step were resolved through discussion with a third researcher from our research team.<br><br>Two independent research teams (SH and BC; SH and EV) conducted the quality appraisal by using the Mixed Methods Appraisal Tool (MMAT) (11, 12). Studies with insufficient methodological quality (answering 'no' to screening questions) were excluded and studies with risk of bias were rated "medium quality". First, they were blinded while appraising the quality of each study, and thereafter discussed their appraisal within the research team, to reach consensus.
Step 4. Data extraction process	For each study, the research team dually drafted one or more CMO configurations, presented as if…(context), then…(outcome), because of…(mechanisms). These configurations described the causal links between context, mechanisms and outcomes (i.e., SAW or WP). From each study, information from the methods, results and discussion section regarding relevant contextual factors or mechanisms leading to the selected outcomes were retrieved. Studies of high quality (see table 2) were used to form CMO configurations. Studies with insufficient methodological quality (answering 'no' to screening questions) were excluded and studies with risk of bias, rated as "medium quality", were only used to support CMO configurations derived from high quality studies.

Step 5. Analysis and synthesis process	We performed analyses by exploring patterns within CMO configurations by thematic analysis, that led to middle range program theories (4). Firstly, after preliminary annotating and extracting data, studies showing similar CMO configurations were coded as barriers or facilitators, regarding each outcome. Those CMO configurations were sorted according to common themes in occupational health and refined in terms of mechanisms. Then, those configurations, categorized in themes, were embedded in a larger chronology of the outcomes, to identify and explain causal effects. Patterns of outcomes (also called demi-regularities) were identified and sorted, using 'if…, then…' statements). Using the thematic analysis leading to demi-regularities, we synthesized mainly from qualitative studies the various mechanisms that occur and under what circumstances those would lead to the outcome. The quantitative studies provided mainly CMO configuration containing of one mechanism, explaining its causal relationship with the outcome. Middle range program theories are based on at least two included studies. In the final stage of the synthesis, we developed an explanatory framework using the initial program theory based on the Capability-for-work model.

On the following pages, first results in detail about studies on Stay at work will be presented, followed by results on Work performance.

CMO configurations for outcome 1: Stay at work

Work outcome: stay at work. Studies that provide insights into the presence or absence of absenteeism have been interpreted in such a way that they generate findings for those who are not absent and thus stay at work.

C = Context
M = Mechanism (resource or reasoning)
O = Outcome
CMO = context-mechanism-outcome configuration
CMHP = common mental health problems
SAW = stay at work

General CMO configurations to explain middle range program theory 1: organizational climate

Middle range program theory 1: A trustful relationship between employee and supervisor, in which the supervisor shows openness to talk about mental health conditions in an open climate in general, may contribute to stay at work among employees with depression, because (a lack of) openness by supervisors is mirrored by employees (13, 14).		
General CMO configurations (demi-regularities)		Facilitators and barriers, based on study specific CMO configurations
Organizational climate	Unhealthy organizational climate, among mostly highly educated employees with depression, (C) contribute to the risk of absenteeism (O), because of the organization perceived as	*Barrier*: an unhealthy general organizational climate (C1), with conflicts and tensions within the team or conflicting values (M), contributes to actual absenteeism (O) among mostly highly educated employees with depression (C) (13).

	inadequate (M1) and conflicts or tensions at the work floor (M2) (13).	*Barrier*: Organization perceived as inadequate (M) leads to dissatisfaction, which contributes to actual absenteeism (O) among mostly highly educated employees with depression (C) (13).
Leadership	If there is an unapproachable supervisor (C) or frequent changes of supervisor (C), then there is an increased risk of absenteeism (O) (13, 14) because the lack of openness among employers (M3) is often mirrored by employees with depression (M2), which leads to untrustworthy relationships (M1).	*Barrier*: A frequent change of immediate supervisor (C) and untrustworthy supervisor (C) result in a lack of trust in relationships (M) which contributes to actual absenteeism (O) among Canadian employees with depression (C) (13).<br>When managers avoid talking about depression (C), this is often mirrored by employees (M), which is a barrier to stay at work (O), among employees with depression from various sectors (14).<br>*Facilitator*: managers offering help for employees with self-reported depression (M), contributes to stay at work, in a work environment where there is openness from managers (C) (14).
Raise awareness		Raise awareness of mental health conditions (M) and challenge stigma, by offering job retention service staff (counsellors or therapists) (M) lead to stay at work among service users with mental health conditions (O) (15).

General CMO configurations to middle range program theory 2: social support

Middle range program theory 2		
Adequate timely social support and supportive relationships increase the chance to stay at work among employees with CMHP, especially among those with stress related problems (16-21), because it helps obtain a manageable workload (16). The most prominent support agent is the supervisor (13, 14). Job support in particular, in which colleagues and supervisors are willing to assist and listen to work related problems, helps to increase the chance to stay at work (16, 17, 19, 20). Facilitation, by either a mental health professional or job retention specialist, who acts independently, with sympathy, pragmatism, who provides an expert insight and who is familiar with the work place improves the likelihood to stay at work (15, 16, 22). This may be because of an encouraging attitude and knowhow about the employment issues.		
General CMO configurations (demi-regularities)		Facilitators and barriers, based on study specific CMO configurations
Social support	If employees with affective disorders receive adequate timely social support (C) and if employees with stress receive supportive communications (C) then the chances of sickness absence decreases (16-21), because of job support, in which colleagues and supervisors are willing	*Facilitator*: If the employee with a stress related disorder receives supportive workplace communications (C), then they can gradually and slowly increase the workload, leading towards full return to work (O), because they inform their supervisor earlier when being overloaded (M) (36) (16).

	to listen (M1) and help with work related problems (M2). Most prominent support agent is supervisor (13).	*Facilitator*: Trusting and long-term relationships (M), or vice versa poor relationships, increases the likelihood to stay at work (O) among those with stress (C) (17, 18). The presence of social support (M) acts as a facilitator to stay at work (O) (16-18), prominently from the supervisor (13), other support actors are not clearly described.<br><br>*Facilitator*: Receiving more job support (M) decreases odds of long-term absenteeism (O) among Dutch workers with an affective disorder (C), (19, 20), and also for stressed employees who are partially on sick leave (C) (16).<br><br>*Neutral*: perceived rewards for the work (M) is not significantly associated with sick leave (O) (23).<br><br>*Barrier*: seeking late for social support (M) is perceived as a barrier to stay at work (O) among male employees with depression, or anxiety disorder or substance abuse/dependence (21).
Facilitation by external professional	If there is contact between the workplace and health care (C) and the employee receives supportive communications (C), then it is easier to fully return to work or stay at work (O), because external professionals have an insight in the workplace (M) and use a person-centred and pragmatic approach (M2) (15, 16, 22, 24)	*Facilitator*: For employees partially on sick leave due to stress related disorder (C), fully return to work (O) is easier when receiving support communication (M1) and contact between workplace (supervisor, colleagues) and health care professional, by facilitation (M2), or through a visit conducted by an external professional, to the workplace and the supervisor by the health care professional (M3) (16).<br><br>*Facilitator*: Trained professional counsellor or mediators effectively deal with anticipated problems or behaviour of employees (C) through an expert insight (M2) and person-centred approach (M3) or by short term coaching (15, 22), may lead to stay at work (24). Mediators (experts in job retention services) who are independent (M1) and encouraging in their attitude, soft in their support characteristics (M2), who know about the employment issues (M3), and who are pragmatic in employment (M4) (15).

General CMO configurations to explain program theory 3: perceived job characteristics

Middle range program theory 3: If an employee with CMHP experiences low job demands, then there is a higher chance to stay at work, possibly because experiencing low job demands helps an individual to exert control over their work (17, 19, 20, 25, 26). However, other studies show that job demands do not influence absenteeism (23, 27). Also, for job control, we cannot discover a clear pattern because some studies show no effect on stay at work (19, 23, 27), while other studies show a positive effect. If an employee experiences high job control, this has a positive effect on stay at work because it creates a sense of control over one's own work, including adjustments that can

be made (15, 20, 25, 26). Heavy work load, overtime/over hours and high job strain reduce the chance to stay at work, among employees with stress or depression (13, 18, 26). Job modifications help to stay at work, however in a different way for white collar and blue collar workers, due to the type of duties that needs to be completed in office hours (16, 17, 28, 29).		
General CMO configurations (demi-regularities)		Facilitators and barriers, based on study specific CMO configurations
Subjective (experienced) job demands	Job demands: Studies showed that experienced low job demands predicts lower sickness absence and experienced high job demands increase the risk of sickness absence, because it allows an individual to exert control over their work (17, 19, 20, 25, 26). Other studies show that the experienced job demands do not influence absenteeism (23, 27).	*Barrier*: Among older employees with psychological problems, among Dutch workers with depression and anxiety, among female government employees and health care employees with psychological distress, and in general among employees with stress (C), the experience of high job demands (M) increases (likelihood of) sickness absence (O) (17, 19, 20, 25, 26).<br>*Neutral*: for employees with stress or a general group of employees with a mental disorder, work stressors such as high demands do not influence absenteeism (23, 27) of which one study removed 4 out of 5 items and therefore presents limited analysis. Also, one study shows that the impact of high job demands is higher on work performance than on SAW (23).
Subjective (experienced) autonomy/job control	Job control, especially making adjustments over one's work (M) seems mostly beneficial and not harmful to stay at work (O) among employees with common mental problems. It remains unclear whether the experience of job control does have an effect on (long term) sickness absence with studies showing no effect (19, 23, 27) and four studies showing an effect, resulting in the ability to exert control over one's own work, including adjustments can be made (15, 20, 25, 26)	*Facilitator*: Among government employees and health care employees with psychological distress (26) (51), employees with depression or anxiety (20) and among older employees with psychological problems (28) (25), higher job control decreases the likelihood of sickness absence (O), because high job control (M1) allows the individual to exert control over their own work, including adjustments that can be made (M2) (15, 20, 25, 26).<br>*Neutral:* For workers with an affective disorder (19, 27), or with mental disorder in general (23) (C), there is no effect on risk of long term absenteeism (O) because of the level of experienced job control (M).
Job strain and job management	Job strain: Heavy work load, overtime/over hours and high job strain (C) contribute to the risk of absenteeism (O) among employees with stress or depression (C) (13, 17, 18, 26), because of the experience of mismanagement of work load (M) (17).	*Barrier:* Among government employees and health care employees with psychological distress (C) high job strain (M) decreased the likelihood of sickness absence (O) (26). Excessive workloads and work hours (C) for clinical psychologist in solo practices (C) contributed to going on sick leave due to burn out (O) (17) or for high SES employees with symptoms of depression (C) to actual absenteeism (13). When employees or self-employed with stress (C) faced mismanagement of work load (M) (17, 26), this reduces the chance to stay at work on the long term (O). Stressed industrial employees mention that high work load contributes to a reduced chance to stay at work (18) (medium quality study).

Job modifications	Job modifications and management of work load by extending work carefully (M) and adjusting after evaluations (M) contributes to stay at work (O) (16, 17, 29, 30) among those with CMHP, but only for white collar workers who are flexible in their duties (C) (28).	*Facilitator*: For employees partially on sick leave due to stress related disorder (C), choosing the right job characteristics (M1) (extending working hours, load) and increasing the workload carefully including adjustments after evaluations (M2), contributes towards a full return to work (O) (16, 17). This is not the case for blue collar workers (28). A problem-solving intervention, in which work environment was adjusted (not described what adjustments) helps to stay at work among Swedish workers who suffered from CMHP or work-stress (30).<br><br>Canadian workers with mood or anxiety disorders (C) required some kind of job modification (M1) to continue working (O) due to chronicity of symptoms, especially those with a concurrent disorder (29), such as changing number of hours, type of work, way to carry out tasks.
Work experience		*Facilitator:* having more work experience (C) decreases the chance to burn out among stressed self-employed (17).
Type of duties	Among employees with distress, having less stress leads to the likeliness to stay at work (O) for blue-collar worker versus the white-collar worker (C). Although blue collar workers have lower levels of responsibilities (M) (26), there is less flexibility (M), that makes it less likely for those employees to stay at work (O) (28).	*Barrier*: Type of work: higher educated work (human service occupations versus lower SEP (maintenance, cooking) (C) leads to more strain (M), leading to higher chance of absenteeism among workers with distress in government employees in public service and health care (26).<br><br>*Barrier*: workers with a high SES had more job strain (M) and as a result a higher risk of sickness absence (O) (26).<br><br>*Facilitator*: (white-collar workers have the ability to work extra hours (evenings or weekends) to compensate for slower task completion whereas, for blue collar workers who experience psychological distress (C), the worksite is not open after normal hours (e.g., construction) or they clock in and out on set time limited shifts (e.g., manufacturing) and therefore experience less flexibility to stay at work due to the type of work (28).

General CMO configurations to explain program theory 4: coping styles

Middle range program theory 4: A lack of adaptive skills, due to reduced psychological flexibility and a diverged range from positive to negative experiences of situations, reduces the capacity to bear responsibilities, which in turn has a negative effect on absenteeism (17, 19, 31). Active coping styles, such as exerting control over one's own work and workload and striking a balance between positive and negative influences of work (16, 17, 25, 32, 33), learning to gain mastery over symptoms (e.g. by control-

ling worrying and catastrophizing) and adjusting and evaluating the workload (16, 25) seem helpful strategies for employees to stay at work. Useful coping and self-management skills are: being more alert, reading and understanding own signals, making adjustments and informing supervisor or colleagues, protecting oneself, taking control, and being assertive (13, 15-17, 24, 25, 34, 35). Also, being highly motivated towards the job increases the likelihood to stay at work (16, 18, 36, 37). Adversely, provisional strategies to compensate shortcomings seem not effective among workers with CMHP (38). Employees with symptoms of depression who do not talk about it or deny symptoms have a higher risk of absenteeism (13, 14, 17, 21). Interventions such as e-health CBT or face to face coaching to improve coping skills help to increase self-management in daily life or decreased symptoms, and consequently contribute to stay at work (22, 34, 36, 39-41). Also, when work was addressed besides personal problems, especially with both employee and supervisor, then there was a higher chance to stay at work because the mismatch between the employee and work environment was addressed (self-management considered as process freedom: freedom to create opportunities within the context) (22, 30, 42).		
General CMO configurations (demi-regularities)		Facilitators and barriers, based on study specific CMO configurations
Adaptive skills as a result of the (diverged) experience of the situation	The 'Big Five' personality characteristics (neuroticism, extraversion, openness, agreeableness and conscientiousness) (C) had no influence on absenteeism (43), but diverged perceptions (less psychological flexibility) (M) and a different experience of situations (M) lead to reduced capacity to bear responsibilities (O), which in turn had a more severe effect on absenteeism (O) (17, 19, 31). Getting more relaxed and less tense (M) helps to approach work differently (M) and therefore helps to fully return to work (O) (16).	*Barrier*: increased conscientiousness as a result of mental problems (M1), less psychological flexibility (M2) and a different experience of situations (M3) impede the capacity to bear responsibilities (O), which has a negative effect on absenteeism (O), among stressed and depressed or anxious workers (17, 19, 31).<br><br>*Facilitator:* being more relaxed (M) and being less tense (M) helps to fully return to work (O) for stressed workers (C) (16). For employees with stress, it seems important to provide (self-guided internet based) tools and strategies to manage symptoms by active coping with various results on stay at work, positive for two studies (36, 40) and no effect on sick leave for one other study (44).<br><br>*Neutral:* among workers with psychopathology (depression or anxiety) (C) there is no association between absenteeism (O) and the 'Big Five' personality characteristics (M) (43).
Motivation and intention to work	Among employees experiencing stress or mood related disorders (C), motivation influences the likelihood to stay at work (O), because of barriers such as low satisfaction before sick leave, wanting another job and facilitators such as a positive attitude, work engagement and readiness/intention to work. (16, 18, 22, 37, 42)	*Barrier*: employees with low motivation or wanting work in another field, for employees with stress related problems (C) are more often absent (O) (18) (medium quality study).<br><br>*Facilitator*: a positive attitude and intention to (return to) work (M) influence the likelihood to fully return to work from part time return to work (O) for employees with stress related disorders (16). For Dutch employees with CMHP, who are mostly highly educated were already in high or stable moderate work functioning, there is a higher chance to fully return to work, because of work engagement and readiness to stay at work after RTW (37).<br><br>Addressed personal and work-related concerns by short term counselling and referral (M), receiving motivational interviewing (M) and being motivated towards positive change by

		trained professionals (M) reduces the amount of working hours missed (O) among employees with depression or anxiety (C) (22, 42).
Have knowledge about condition, signals and care	If employees understand mental health by having knowledge about relationship between physical and psychological symptoms (C), then this leads to an increased chance to stay at work (O) and enter of treatment (O), because of being more alert, reading own signals and knowing own roles (M) (13, 15, 17, 35, 42)	*Barriers*: Being confused about relationship between their physical symptoms and psychological ill-being) (M) contribute to absenteeism (O) (9) (13).<br><br>*Facilitator*: By educating employees about conditions and self-care (C), employees will be alert to signals and will understand mental health (M), leading to stay at work (15, 17, 35)<br><br>To know own roles (M) and an increased ability to read signals (M) can prevent overload and therefore prevent burnout (O) (17).<br><br>To educate employees with depression to enter treatment helps to stay at work (35, 42).
Coping and self-management	Employees with stress or depression (C) who have learned to exert control over their own work, by 1) balancing positive and negative influences of work , 2) keeping enough 'down time' and 3) making adjustments and informing colleagues (M) are more likely to stay at work or prevent burnout (O) (15-17, 25, 33). Because of workplace stigma, employees with depression pass as normal/façade and hide their 'true' selves to continue working (33). If employees with stress mismanage workload then there is a lower chance to stay at work or fully return to work (O) because there is an inability to protect oneself (M) (16), or they have a reduced capacity to bear responsibilities (M) (17, 26). Employees with depression or anxiety, using periodically compensatory strategies for shortcomings were helpful on the short term, but do not last on the long term to stay at work (38).<br><br>Counselling and coaching interventions, through CBT-e-health and face to face (C), in which employees increase self-management skills by problem focused coping, learning new strategies, addressing work-related concerns besides personal or	*Facilitator:* Employees with stress (C) who have learned to exert control over their own work (M) and striking a balance between positive influences (finances, distraction, maintaining routine and purpose) and negative influences (e.g. work stress) (M) are more likely to stay at work (O) (15-17, 25, 33)..<br><br>*Facilitator:* Learning to make adjustments after evaluating workload (M) among employees with CMHP (C) helps to stay at work or increase hours to return to work (O) (15, 16, 25)..<br><br>*Facilitator*: Addressed work, besides personal or mental health related concerns in short term counselling and referral (M), receiving motivational interviewing (M) and being motivated towards positive change by trained professionals (M) reduced work hours missed (O) among employees with CMHP (C) (22, 30, 42).<br><br>*Facilitator*: To become more assertive towards colleagues and supervisor (M) and to inform supervisor earlier when feeling overloaded (M) results in increased self-efficacy (M) and decrease of complaints, which helps to gradually fully return to work or stay at work (O), among employees with stress, depression or anxiety (C) (16, 34).<br><br>*Facilitator:* managing self-discoveries around authenticity, being yourself or passing as normal and hiding their true selves, was used to continue to perform at the workplace to anticipate workplace stigma among depressed workers (33)<br><br>*Facilitator*: for female health care workers with elevated level of stress, nature-based program helped to reduce sick leave by learning strategies for managing stress (M1), setting limits of

	mental health related concerns, lead to a decrease of complaints (O) and subsequently to less absenteeism (O) (15, 22, 24, 34, 36, 39-41). Especially when lead by a consultant talking to both the supervisor and the employee (30), because of goal setting, behavioural change, involving supervisor, which helps individuals to take control, advocate, and develop assertiveness skills (M).<br><br>No effect was found for mailed advice (44) for absenteeism, or by an IPS-based intervention for employees with stress, including coaching on symptoms and coping skills (45). Also, no effect was found for problem-based therapy in mild depression or employees with CMHP who are still at work (45, 46).<br><br>Studies reported that counselling or CBT may lead to reduced sickness absence among employees with depression through increased coping, however mechanisms were not clearly reported (32, 35, 41, 42, 46).	engagement M2, in nature (C) (36). For Australian and English participants with depression, having enough 'down time' helped to stay at work (33)<br><br>*Barrier:* when employees or self-employed with stress (C) faced reduced capacity to bear the responsibilities (e.g. for health care provider: putting clients' needs first due to human service occupations) (M), this reduces the chance on the long term to stay at work (O) (17, 26).<br><br>*Barrier*: for employees partially on sick leave due to stress related disorder, fully return to work is limited when the person has difficulty of protecting oneself and current decreased work capacity) and solutions (16).<br><br>Neutral: Individual Placement and Support (IPS)-based intervention, containing of job coaching was specifically tailored toward each employee's problems (M1) and the current individual job situation (M2) and use of goal setting (M3), has no effect on symptoms of depression and coping skills for employees who felt mentally distress due to work-related issues (C) (45).<br><br>*Barrier:* Among employees with mild depression or stress (C), no effect on stay at work (O) was found for problem-based therapy or activating coping skills in preventative face to face counselling (M) (45, 46).<br><br>However, a positive effect was found among workers with depression in e-health (CBT) interventions and face to face coaching (34, 39, 41). A problem-solving intervention was effective when a trained consultant had meetings with both manager and employee (30).<br><br>Forcing the work role (just doing it) (M1) and compensating for shortcomings (M2) (by skipping breaks, working longer or continue work at home, using this periodically), warding off work strain (grin and bear it) (M3) are unconscious ways to keep working (O). Those strategies were used periodically but not sustainable and do not help on the long term to stay at work, among Swedish workers with depression or anxiety (C) (38).
Disclosure	Employees with symptoms of depression are more at risk of absenteeism (O) when not talking about it (M), resulting from the relationship with supervisor or the supervisors' avoidance to talk about it (C). (13, 14)	*Barrier:* the relationship with supervisor (C) results in a lack of disclosure of one's condition and needs (O) (13). The avoidance of talking about an employee's condition by managers (C) was mirrored by employees (M2) both resulting in a higher risk of absenteeism (O) (14).

Deny symptoms	If employees deny signals (9) (13) and show avoidant behaviour (14), then they are more likely to stay at work on the short term (positive effect on stay at work), but this does not protect them from absenteeism on the long term, because of late seeking for support (21).	*Barrier*: Those who deny signals, (M1) (13), who show avoidant behaviour (M2) (14) are less likely to stay at work (O).<br>*Barrier*: employees with major depressive disorder, dysthymia, simple phobia and substance abuse / dependence (C), have an increase likelihood to sickness absence (O) because they are less likely to seek for help and therefor late reception of social support (M) (21).

General CMO configurations to explain middle range program theory 5: health symptoms and severity

Middle range program theory 5: Experiencing better health contributes to stay at work, because the experience of lower severity of symptoms leads to less impediment in work performance (via increased cognitive functioning, increased productivity or decreased exhaustion) (20, 27, 47-50). Likewise, facing other health complaints (comorbidity resulting in fatigue) as well as previous sick leave, result in a lower chance to stay at work (31, 47, 51, 52). Interventions on symptomatology (M) (either by pharma therapy, psychotherapy or combined) reduce the severity of symptoms (O). This outcome becomes a contextual factor for some other mechanisms such as experienced work performance on the outcome SAW. Interventions offering psychotherapy or pharma therapy, for employees who currently experience symptoms, seem more effective in enabling employees to stay at work, than preventative treatment or stress reduction interventions (24, 35, 36, 39, 41, 44-46, 53-56).		
General CMO configurations (demi-regularities)		Facilitators and barriers, based on study specific CMO configurations
Theme		Explanations using CMO configurations
Self-reported health	If employees with CMHP experience better health, by good self-rated health (C), this contributes to stay at work (O), because of the experience of lower severity of symptoms (M) (20, 27, 47-50).	*Facilitator:* Employees with depression rating a relatively good self-assessed mental health (C) (45, 47) or a better physical health (C) (27), have a higher chance to stay at work and vice versa (45), due to the experience of lower severity of symptoms (M) (47). This could differ among male and female, where female depressed employees are less likely to stay at work when having moderate-severe complaints and male are less likely to stay at work when having mild complaints (27, 48, 49).<br>*Barrier*: having poorer general physical health among depressed workers (20, 27, 48, 50) (C), or the experience of exhaustion (M) among those with stress (C) limits to stay at work (O) (17), more than job characteristics (20, 27).<br>*Barrier*: Increased severity of depression (M) influence absenteeism (20, 47))
Interventions to reduce symptoms	Interventions on symptom reduction (either by pharma therapy, psychotherapy or combined)	Symptom reduction deems to be a major resource in several intervention studies for employees with depression, increasing the chance to stay at work or prevent absenteeism (pharmacotherapy, psychotherapy or combined), both in controlled and uncontrolled studies, and especially employees with

	aim to reduce severity of symptoms and therefore increase the chance to stay at work, possibly through improved work functioning. However, through which mechanisms the relationship between severity of symptoms and stay at work operate stays unclear. Possibly through reduced stress, consultation that changes coping styles, in which psychotherapy or pharma therapy seems more effective than interventions reported as preventative treatment (24, 35, 36, 39, 41, 44-46, 53-57).	depression (32, 35, 41, 42, 53, 57). Preventative programs (targeting those at risk of absenteeism with psychosocial health complaints) aiming to reduce symptoms, such as mindfulness, but did not lead to sustainable Stay at work, among those with depression or psychological complaints (24, 46, 54). Among those with anxiety only two studies were found with treatment, face to face coaching and dog services for veterans with PTSD, to reduce symptoms which seemed effective to reduce absenteeism (39, 55).<br><br>American workers with any sleep disorder, with shift work as a risk factor, have more missing work time and absenteeism, possibly because they struggle in terms of work performance (concentration, organization at work, making mistakes, impatience, avoiding interactions with co-workers (56).<br><br>A nature-based stress management course (C) reduced sick leave through stress reduction by using nature to recover from stress (36). Mailed advice about relaxation techniques, to reduce stress had no effect on sick leave among distressed employees (44).<br><br>An IPS-based intervention aiming to retain employees in the job, containing of job coaching, had no effect on sickness absence and had no effect on symptoms of depression, exhaustion and coping skills, for employees who felt mentally distress due to work-related issues (C). The intervention was specifically tailored toward each employee's problems and the current individual job situation (M1) and use of goal setting (M2) (45).
Comorbidity (other health problems resulting in fatigue)	If an employee has comorbidity besides other CMHP, such as insomnia (C), then it is harder to stay at work (O), because of is less psychological flexibility (M) and fatigue due to a lack of sleep (M) (31, 47, 51).	*Barrier:* For patients with symptoms or syndrome of insomnia, having the syndrome of insomnia, rather than symptoms or no insomnia (C), leads to higher absenteeism (O), due to reported health problems and fatigue (M) (51).<br><br>*Barrier:* for depressed or anxious employees (C), who also suffer of insomnia seem less likely to stay at work (O), because it is hard to cope with sleep disturbances due to mental illness, and less psychological flexibility (M) (31).<br><br>*Facilitator*: Those having no comorbid disorder (C) face a lower chance of absenteeism (O), among employees with depression (C) (47).

General CMO configurations to explain middle range program theory 6: personal context

Middle range program theory 6: Socio-demographic characteristics, as owning a house or being married, may contribute to stay at work, based on possible underlying mechanisms such as financial drive or being provided with social psychological and economic resources (17, 20, 26, 47). Employees with CMHP who had more life events, personal problems or exposures in other life domains than work may experience tension or confusion about symptoms, which leads to more absence days (13, 17, 26, 58).		
General CMO configurations (demi-regularities)		Facilitators and barriers, based on study specific CMO configurations
Life events/life stressors or personal problems	If employees with CMHP (C) have had more life events or non-work exposures or personal problems (C), then this leads to more absence days (O), because of confusion or tension (M) (13, 17, 26, 58).	*Barrier:* more life events/life stresses/non-work exposures reduce the chance to stay at work (O) among people with CMHP (17, 26, 58).<br>*Barrier*: when experiencing a personal problem (marital problems, money issues, death injury of close family member, or particularly demanding situations) (C) this leads to confusion of symptoms (M) reducing the chance to stay at work (O) (13).
Private situation and socio demographics	Measured socio-demographic characteristics (marriage, owning a house, being self-employed) (pre-existing, context) are possible stimuli to stay at work (17, 20, 47). If an employee is being married then this may lead to continue working because of provided social psychological and economic resources (47) and the financial drive due to lack of insurance or having financial obligations or strain (17, 20).	*Facilitators*: owning a house, being married, being self-employed (C) are found to be possible stimuli to continue working (absence of absenteeism (17, 20, 47). (O), for employees with depression or stress related symptoms (C), because the job provides social psychological and economic resources (M).<br>Neutral: Those with no school education compared to those with education (C) have no significant higher chance on absence of absenteeism (O) (47).
Previous sick leave		*Barrier*: The chance on recurrent sick leave reduces (O) when having less episodes of sick leave in the past among employees with common mental disorders (C) (52).

General CMO configurations to explain program theory 7: Features of interventions to Stay at work

Middle range program theory 7: If interventions focus on multiple components (medication combined with psychotherapy, self-treatment combined with offered program or several approaches in psychotherapeutic support), in which interventions target both personal inputs (symptom reduction and coping with symptoms) and work inputs (coping at workplace or a better work related health), this may lead to an increased likelihood to stay at work (15, 32, 34-36, 39-41). In these interventions, the use of

online or telephone support systems (context), always in addition to individual care or coaching by a case manager or therapist, seems effective because it increases adherence, better access to treatment and early and regular screening and it tailors messages and integrates learned skills into daily life (34, 35, 40-42). However, it remains unclear how this influences the employee's working life. Preventative, worksite based, job retention interventions or adding a work-focused intervention to integrated care did not seem effective on the outcome of stay at work, in the phase that employees are still working (24, 45, 46, 59).		
General CMO configurations (demi-regularities)		Facilitators and barriers, based on study specific CMO configurations
Multi component targeting personal inputs and conversions	Multi-component interventions (C) (medication and psychotherapy, or several approaches in psychotherapeutic support or self-treatment combined with offered program) may help to stay at work (O) because those interventions target personal inputs (M), and because interventions are tailored online and transfer learned lessons to home situation/daily life (M) (15, 32, 34-36, 39-42). Preventative, worksite based, job retention interventions or adding a work-focused intervention to integrated care did not seem effective on the outcome of absence of absenteeism (24, 45, 46, 59). There is a lack of evidence towards interventions targeting both the individual worker and the work environment.	*Facilitator*: multi-component tailored interventions (medication and psychotherapy (32, 35, 42), or several approaches in psychotherapeutic support (15, 36, 39-41), seem to decrease absenteeism by reduction of existing mental health complaints or conditions (M1) or improving self-management and coping skills (M2) (34, 40), which has a positive effect on stay at work (O).<br><br>*Facilitator:* Elements to make treatment more effective seem the combination of face to face treatment and a self-guided internet program (40, 41), transfer the learned lessons to home situation/daily life) (35) and use of expertise of case manager involving both the employee and supervisor/manager (15, 30, 42) resulting in an increased chance to stay at work.<br><br>*Neutral:* Worksite based programs, focused on prevention of sick leave due to psychosocial conditions by face to face individual coaching and three way consultation (24, 45, 46) had no effect on sickness absence, possibly because there was already a low prevalence of sickness absence since employees were 'at risk' but not yet reported sick (24, 46).<br><br>*Neutral:* An individual job retention intervention (IPS-based), containing of job coaching was specifically tailored toward each employee's problems (M1) and the current individual job situation (M2) and use of goal setting (M3). This has no effect on symptoms of depression and coping skills, for employees who felt mentally distress due to work-related issues (C) (45). Adding a work-focused intervention to integrated care among American veterans with depression has no effect on stay at work (59).
Technology to increase quality of care and integrate learned skills into daily life, in addition to personal care and assistance.	Telephonic outreach and care management programs educate employees to enter treatment (psychotherapy and/or antidepressant medication) (35, 42) or enhance regular monitoring of treatment by health care service, which leads to improved work-related health rather than absence of absenteeism. This occurs because technology provides structure to improve adherence and regularity of screening	*Facilitator*: Self-guided internet based and mobile supported stress management program has no significant effect on absenteeism but it has an effect on work related health, due to higher grade of interactivity (M1) and simplicity (only 2 components addressed, problem solving and emotion regulation techniques) and mobile components which foster the transfer of training components into daily life (M3) (34, 40, 41).

	by the use of interactive sessions supplemented with online tools (40-42). Also, it helps to integrate learned skills on symptom management into daily life (40, 41). However, technology is always used in combination with or in preparation to personal contact with professionals, such as employee-assistance programs or regular care (34, 41, 42).	*Facilitator:* A supporting self-treatment app based on CBT (C) is most effective when being integrated and implemented through employee-assistance programs (M), among employees with depression to reduce absenteeism (O) (41, 42).<br><br>*Facilitator:* If telephone outreach is offered on top of employee assistant programs, this leads to lower absenteeism among American employees with moderate or major depression, because of regular contact by person or by phone helps for follow up and reassessment among patients with major depression in the USA (35, 42).

CMO configurations for outcome 2: Work performance

Work outcome: work performance. We emphasize on factors and mechanisms that relate to the outcome of presenteeism (being at work whilst sick), reduced or impaired work performance and work functioning.
C = Context
M = Mechanism (resource or reasoning)
O = Outcome

General CMO configurations to explain program theory 1 Social support

Middle range program theory 1: A work environment where managers feel comfortable to offer help and support employees, helps these employees to feel motivated and valued, which in turn has a positive effect on their job performance (14, 60). Practical job support from colleagues and managerial support from supervisors, offered continuously while functioning at work despite CMHP, helps to improve work performance, because of trust and empathy received by the employee (14, 20, 33, 37, 38, 58, 60).		
General CMO configurations		Facilitators and barriers, based on study specific CMO configurations
Theme	Demi-regularity (pattern)	Explanations using CMO configurations
Managerial support	If an employee with CMHP receives support from managers (which managers learned through training about dealing with mental health issues, and an open culture (C)), then there was a positive effect on job performance, because of offered modifications (M1), comfort talking about condition for managers (M2) and the employee's experience of being motivated and feeling valued (M3) (14, 60).	Facilitator:<br>- Support from managers (when managers offered help to employees) (C) had a positive effect on job performance (O) in 8 different countries for employees with depression (C), because it created openness and less avoidance to talk about mental health with employees (M1) and it helped to remain motivated and feeling valued (M2) (14).<br>- Trained managers who have received support and training in dealing with mental health issues (C) were more likely to recognize and act on problems earlier (M) and their openness was mirrored by employees (M), feeling comfort to talk about depression with managers (14).<br>- Managerial support helps to increase work performance, because supportive managers offered work modifications, reduced hours, redeployment, manageable workload, being supernumerary (M) (60).
Social support at work	Continuous job support helps to improve work performance (O) through job accommodations (M) and has the potential to increase a sense of belonging and coherence (M), which seems not the case in a competitive work environment (C) (58). This support can	Facilitator:<br>- Getting job support (M) and continued psychological and work support decreases chance of reduced and impaired work performance (O) for Dutch workers either employed or self-employed (5%), with depression or anxiety disorder (C) (20, 37).<br>- Disclosure to receive support from the manager and co-workers among employees with depression or anxiety syndrome, helps to keep working and among employees with depression, it has the potential to create a sense of belonging and coherence as well as empathy and trust among

	be practical while still at work from colleagues (C) and it can be job accommodations or reduced work load offered or supported by manager, leading to received empathy and trust from colleagues/supervisor<br><br>(14, 20, 33, 37, 38, 58, 60, 61).	colleagues and managers, (33, 38). Also, if psychological and work support is continued after returning to work, then employees with CMHP experience more successful work functioning (37).<br>- If the worker is in a highly competitive work environment (C), then work-related social support (M) from superiors and colleagues may be less, leading to role limitations and social functioning limitations, among employees with depressive disorders in Taiwan (58).<br>- Identifying workplace support by offering job accommodations and practical support by managers or colleagues (C) helped to increase work performance (O) among employees with depressive disorder or dysthymia or anxiety. Colleagues are providers of practical support for employees who are working despite CMHP (M) (60, 61).
Support by general practitioner		Neutral:<br>- On top of regular treatment, regular self-screening and enhancing the possibility to discuss the impact of symptom lapse on ones work ability during GP visits for patients with mild to moderate depressive disorder (C), did not increase work ability on the long term but on the short term (O) it leads to higher uses of antidepressant (M1) and higher social support in a subacute phase (3 months) (M2). Screening and consultation by the GP lead to higher uses of antidepressant and higher social support, patients may have been more open about their depression at work and thus to receive more support from colleagues and managers (62).
Rewards		Barrier:<br>- If the worker is in a highly competitive work environment (C), then rewards in terms of respect and support from superiors and colleagues may reduce (58).<br>- Among Dutch employees with a mental disorder, the experience of lower rewards (whether the organization gives the reward he or she deserves for the work), is associated with lower work ability (23).
Context		Support offered from workplace or from health care system:<br><br>psychotherapeutic consultation in the workplace (C), for men using psychotherapeutic consultation, is similarly effective as consultation in outpatient clinics (C) on the employees' workability (63) .

General CMO configurations to explain program theory 2: perceived job characteristics

Middle range program theory 2: the combination of high job demands and low job control may reduce work performance among employees with CMHP, because of struggling to manage time and performing interpersonal and output tasks (23, 27, 61). However, other studies show that facing higher job demands or lower job control does not increase the chance of reduced or impaired work performance (20, 64).

Theme	Demi-regularity (pattern)	Explanations using CMO configurations
Job modifications		Facilitator:<br>- Employees with anxiety or depression (C) who get offered work modifications by their managers (M1), reduced hours, deployment, to enable a manageable workload, or being supernumerary, felt this helps at work (60).
Subjective (Experienced) Job demands and job control	Among employees experiencing depression (C), psychologically demanding work (M) and low job control lead to an increased difficulty with managing time and performing mental-interpersonal and output tasks (O). However, facing higher job demands or lower job control in one study does and in two other studies does not increase the chance of reduced or impaired work performance, possibly because of diminished emotional reactivity (20, 23, 27, 61, 64).	Subjective (experienced) job demands<br>Barrier:<br>- Dutch health care employees with a mental disorder or American employees facing depression, who experience high psychosocial work demands, show lower work ability (23, 27).<br>Neutral:<br>- Among Dutch workers who have an employer or who are self-employed (5%) with depression or anxiety disorder, as well as among Korean workers with depression (C), the chance of reduced and impaired work performance (O) is not affected by the experience of higher job demands (M), because of diminished emotional reactivity (20, 64).<br><br>Experienced job control<br>Barrier<br>- Experienced lower job control (M) increased the amount of difficulty workers had managing time and performing interpersonal and output tasks or lower workability, (O) among employees with a mental disorder (C) or depression (C) (23, 27, 61).<br>Neutral<br>- Among Korean workers with depression (C), the experience of higher job control (M) is not associated with reduced work performance but among Dutch employees with and without psychopathology job control is associated with impaired work performance (O) (20, 64).
Objective job demands	A higher number of working hours is not associated with impaired work functioning but is significantly associated with reduced work performance (20).	Neutral:<br>- A higher number of working hours (M) is not associated with impaired work functioning but is significantly associated with reduced work performance, among workers with depression or anxiety disorder (20).

General CMO configurations to explain program theory 3: Coping styles

Middle range program theory 3: If employees experience reduced capacity to work, they initially use working façade strategies (such as increasing hours or taking work home) to compensate possible shortcomings and to avoid reduced performance. Employees use these working facade strategies because of fear and perceived stigma, but these strategies are counterproductive, as they results in emotional exhaustion, fear, dissatisfaction and loss of refuelling on the long term (28, 38, 60, 65). Perceived stigma and sense of loneliness may discourage the person to disclose the mental health problem and trigger to use façade (60, 65). Interventions (e.g. cognitive based therapy, counselling, tailored coaching, employees assistance programs) promote work performance because they improve useful coping styles, which are 1) to reconsider one's attitude to work (their approach to work tasks, responsibilities and achievements), 2) to reach out for managerial support, 3) to learn new approaches to manage job de-mands, 4) calming the mind and retrieving space (22, 27, 38, 40, 41, 45, 66-68). Preventative interventions have no impact on work performance (24, 62).		
General CMO configurations		Facilitators and barriers, based on study specific CMO configurations
Theme	Demi-regularity (pattern)	Explanations using CMO configurations
Short term coping	If employees with stress, depression of anxiety use short term coping, such as façade strate-gies, increasing time at work, taking work home, then those cramped or even desperate measure lead to prevent or avoid reduced per-formance. However, this type of coping does not last long, because it results in fear, dissat-isfaction and loss of refuelling on the long term (28, 65).	Barrier:<br>- Employees (blue collar workers only, not for white collar workers) under stress (C) may extend their time at work (M1) or take their work home (M2), thereby ensuring that tasks are completed on time, thus avoiding reduced performance (28).<br>- Persons with CMHP, mainly women in regular job market, with depression and anx-iety (C), use working façade, which resulted in fear (M1), dissatisfaction (M2) and loss of refuelling (Mresponese2), use those strategies to handle reduced capacity to work on the short term (O), however this was not effective on the long term and led to exhaustion because it was considered energy consuming (65).
Disclosure and stigma	If employees with anxiety or depression feel stigmatized or lonely, then this will reduce work performance, because they are less will-ing to tell people and instead use working fa-çade (14, 60, 65).	Barrier:<br>- If employees with anxiety or depression feel stigmatized (C), then they are less will-ing to tell people about their illness (M), leading to reduced work performance (60). If managers of employees with CMHP are trained about mental health, it creates openness and comfort in talking about depression (14).<br>- If there is perceived stigma of CMHP, then a sense of loneliness seemed to develop (C), leading to working façade (M), which is used to overcome reduced capacity to work (65).
Coping styles adhered to cope on long term.	If employees with CMHP use rather long term coping styles in their reasoning and reaction,	Facilitators:<br>- Reflexive adaptation by considering one's attitude to work (M), modify work frame

	they increase or improve on work performance, because of the use of reflexive adaptations, reconsidering their attitude to work (approach to tasks and responsibilities), reaching out for support, and learning new approaches to manage job demands as well as stress (27, 38, 61).<br><br>If work performance is already reduced/lower (24), then resources or interventions have a positive effect on work performance and the experience of work, because of peer coaching, learning tools for managing stress (36), addressing personal and work related concerns in counselling (22), counselling to change dysfunctional patterns into new habits (36), reviewing cognitive and behavioural sills (66), making a self-care plan, and reviewing thoughts (66, 67), goal setting (45), tailored by technology with personal feedback and motivational exercises (40, 45, 68).<br><br>Additional interventions on top of Employee Assistant Programmes, such as t-CBT did show mixed results, but had mostly a positive effect on work performance (22, 36, 41, 66, 69).<br><br>Worksite based and preventative programs (all containing of 8-12 personal coaching sessions to improve coping styles) show a positive effect on work-related success among employees with mental distress, however not on presenteeism and psychosocial work characteristics (24, 45, 66).	(M), reach out for managerial support and from co-workers (M) seemed to lead to a sense of belonging and coherence, as well as learning to act on personal cues (M) all enhancing work performance (O) (38, 61). The same employees recuperate from work (M1) by taking moments in a private space, taking at home alone time, to increase fitness and calm the stressed mind, but also to retrieve space and ensure room to manoeuvre (M2), to reduce stress (O) (38).<br>- Manage job demands by learning new approaches (M3) on performance of mental-interpersonal, time management, output and physical tasks, helped to experience less work limitations (27, 38).<br>Resources (interventions):<br>- Nature based stress management course (12 weeks) (C) increased work productivity by learning to use tools and strategies for managing stress (M), and (after one year) changes dysfunctional patterns to develop into new habits (M) (36)<br>- Worksite based Employee Assistant Programme (EAP) intervention for workers with common mental health problems reduces presenteeism because the employees' personal and work-related concerns are addressed (M), short term counselling and referral and use of motivational interviewing towards positive change, which reduced symptoms (O →C) that then increases productivity (C) (22).<br>- Supporting self-treatment app, implemented through employee assistance programs (M), among employees with depression (C), reduce productivity loss (41).<br>Neutral:<br>- T-CBT on top of EAP for Japanese full-time workers with minor depression (C) did not improve presenteeism, despite the fact that for those who cannot readily visit health care services on weekdays T-CBT is offered (66). Mechanisms such as reviewing cognitive and behavioural skills, doing homework (67), creating a self-care plan and reviewing thoughts, did not make a difference.<br>- Goal setting has an effect on psychosocial conditions of employees in the workplace but not as part of preventive coaching (24, 45).<br>- Tailored coaching: Problem focused stress management, tailored by technology with personal feedback and motivational exercises (40, 68, 69) or job coaching (IPS based) by the workplace, tailored towards employees problems (22, 45) helped to increase work performance when problems already existed (struggling at work), but not for behavioural change as part of preventive coaching (24).<br>- Preventive coaching, for employees in health and educational sector with psychosocial health complaints (C) has no effect on psychosocial work characteristics (O), such as fairness, career possibilities and conflicts, based on sessions applying goal setting, behavioural change, involving supervisor and three way consultation (M)

		(24).<br>- On top of regular treatment, regular self-screening and enhancing the possibility to discuss the impact of symptom lapse on patient's work ability (M) with GP visits for patients with mild to moderate depressive disorder (C), did not increase work ability (O). Thus, mechanisms such as regular self-assessment, enhancing possibility to discuss impact of symptoms (M) on patients work ability (O) were of no effect on the long term; only on the short term, because of consultation with GP leads to higher uses of antidepressant and higher social support in a subacute phase (3 months) (62).

General CMO configurations to explain program theory 4: symptom severity

Middle range program theory 4: Self-rated health and severity of symptoms are important predictors of work performance among those with depression, anxiety or sleep disorders, because once the employee experiences less symptoms, work productivity improves (27, 48, 50, 56, 61, 70). Interventions aimed at the individual to reduce symptoms results in increased cognitive functioning, a pro-active attitude towards change, better mental-interpersonal task performance, improved time management and output, which all together lead to increased work performance (22, 32, 35, 36, 40, 42, 45, 54, 57, 67-70). Among those employees with stress, interventions improve stress recovery and symptom management, which subsequently leads to improved productivity (36, 40, 54). Chronicity of CMHP has been showed to reduce work performance (20, 37, 50, 71).		
General CMO configurations		Facilitators and barriers, based on study specific CMO configurations
Theme	Demi-regularity (pattern)	Explanations using CMO configurations
Self-rated health	If an employee has a higher self-rated (mental or physical) health (C), then he or she improves in symptoms (M) and faces less reduced work performance (O) (27, 48, 70).	Facilitator:<br>- Depressed working patients reporting good to excellent health status (C), show relatively less productivity loss (O) because of reduced symptoms (M) among American patients, of whom the majority is female, middle-aged, white, married and has at least college education (70).<br>Barrier:<br>- Employees with depression (C) had an increased amount of difficulty in managing time and performing mental-interpersonal and output tasks (O) when they faced reduced physical health (M) (27) or when they rate their mental health average to fair/poor (M) (48) (48), among American (29) (27) and Colombian (48) employees.
Severity of symptoms and symptom improvement	There is a ripple effect as follows:	Facilitator:<br>- If symptoms improve (O), by receiving usual care including anti-depressants among employees or self-employed individuals with depression (C), then work

	Interventions such as use of medication (32, 50, 53, 57), (telephone) psychotherapy and counselling (22, 32, 42, 45, 56, 57, 67-69), or combined for those with depression (35) and stress reduction programs in nature or using eastern practices (36, 54) for those with elevated stress levels, help to reduce symptoms (O). Reduced symptoms (Outcome → C) lead to the following reasonings (M): improved (cognitive) functioning, improved performance in mental-interpersonal tasks, time management and output (M). Those reasonings are facilitated by counselling, in which identification of roadblocks, addressing work-related concerns and moving towards positive change (M) lead to increased work performance (O) and reduced work limitations (O).<br><br>Work productivity is still reduced among employees with depression compared to healthy controls (32).<br><br>Stress reduction programs increase productivity and reduce presenteeism (O), both by stress recovery and symptom management (M) (36, 40, 54), mostly among health professionals (C) with elevated stress levels.	limitations reduce (O), because of improved performance of mental-interpersonal, time management, output and physical tasks (M) (27, 61, 70).<br>- For employees with chronic or recurrent depression (C), overall work productivity increases (O), due to the use of antidepressant (32, 53, 57). Explanations for the perceived improvements are: reduced symptom severity (M) (32), change of cognitive functioning (M) (56, 57), an underestimated past performance due to current negative view of self (M), or the successful treatment of their subclinical depression (C) (32).<br>- Among workers with CMHD (C), work functioning reduces (O) because of the severity of symptoms, occurring at work (C) (50, 56, 61).<br>- Counselling (psychotherapy, CBT, e-counselling) (C) in general decreases severity of symptoms (O →C) and as a result decreases work impairment (O) and presenteeism and it also increases the experience of work-related success (O) (22, 32, 42, 45, 57, 67-69). For example by identification of road blocks, behavioural experiments, psychoeducation, balancing experiment (69) or addressing work-related concerns, and motivating towards positive change (22) (M).<br>- Employed patients with major depression (C) improves on productivity (O) because of a combination of guideline provided medicine, telephone reassessment, given education and homework assignments (M) that lead to lower depression severity (O →C), therefore leading to improved productivity (O) (35).<br>- Learning emotion regulation techniques (M) (40) reduces presenteeism, among employees with elevated stress levels, mostly women and medium or high educated and health professionals (C) by learning stress reduction techniques, possibly in nature (breathing, relaxing) (36) or through Eastern practices as mindfulness and meditation (54), in order to change dysfunctional patterns.
Comorbidity	If an employee with depression or anxiety faces sleep disturbances (C), then they seem to face presenteeism more often (O), because it is harder to cope with sleep disturbances and they experience less psychological flexibility and more health problems (M) (31, 51, 56).	Barriers:<br>- Dutch depressed or anxious employees working at least 8 hours a week, show that sleep disturbances (C) were negatively associated with presenteeism (O), because it is harder to cope with sleep disturbances (M1) and they experience less psychological flexibility (M2) (31)<br>- Having a sleep disorder (C), more than only having symptoms or being a poor sleeper, lead to significantly reduced productivity (O) due to reported health problems, problems in concentration and organization and relating to co-workers, caused by fatigue, among Canadian patients with symptoms of insomnia or insomnia syndrome (51), because of low sleep quality (56).

Chronicity	Chronicity of CMHP has been showed to reduce work performance, and needs attention in order to maintain work performance (20, 37, 50, 71).	Barrier:<br>- Among workers with CMHD, work functioning reduced (O) because having a chronic depression (C) or lifetime diagnosis of major depression (50, 71).<br>- Among Dutch workers who have an employer or who are self-employed (5%) with depression or anxiety disorder, chance of reduced and impaired work performance increases due to remitted diagnoses (C) (20).<br>- To be fully functioning back at work, patients' family, co-workers or supervisor should have realistic recovery expectations, because CMHP patients who have returned are still struggling with mental health and work functioning problems (37).

General CMO configurations to explain middle range program theory 5: Features of interventions

Interventions that use technology, by providing personal feedback through email, phone or an app may reduce symptoms as well as prevent work limitations. The use of those technologies helps to monitor employees, by tailoring the interventions with personal feedback, fostering changes in belief, reduction of symptoms, facilitating the transfer of training components to daily life and assisting to overcome distance that might reduce treatment adherence (40, 42, 59, 62, 67-69, 72).		
General CMO configurations		Facilitators and barriers, based on study specific CMO configurations
Theme	Demi-regularity (patterns)	Explanations using CMO configurations
Technology	Use of technology in interventions either to reduce symptoms or to prevent work limitations, help to monitor employees, by tailoring the interventions with personal feedback to change beliefs and attitudes and to overcome distance that might reduce treatment adherence (42, 62, 67-69, 72)	Facilitators:<br>- Technology is used to enable tailored interventions, with personal feedback (68)<br>- Interventions that offered communication by telephone or through internet can monitor or possibly increase adherence because of systematic screening, use of professionals as case managers and regularly planned care supervision (42, 62, 67)<br>- Combination of personal health technologies and 3 psychologist-assisted group meetings among male workers with stress and mood problems, has a positive effect on work ability because of personal/tailored health tech based psychological intervention with personal feedback, group meetings and its effect on symptom severity (for depression or insomnia), because those tailored feedback comments change dysfunctional beliefs, attitudes and severity of symptoms (68, 72). Distance can be covered by telephone/e-health programs also, increasing the use of medical health care (42, 69).
Interactive, person centred interventions	Person-centred feedback by app/mail or phone technology enhances interactivity, especially in interventions to reduce symptoms or improve	Facilitators:<br>- Among employees with stress or mood problems and among veterans with depression, e-health interventions facilitate the transition of elements from training to everyday life,

	coping, fostering the transfer of training components to daily life (40, 59, 67, 68, 73).	thanks to increased interactivity, flexible person-centred care, regular application of exercises, homework assignments, reminders, or even additional telephone consultation by a psychologist (40, 59, 67, 68, 73).<br>Neutral:<br>- One intervention reports that providing feedback on booklet and giving reminders improved functional impairment, but not work ability (67).

References:

1. Pawson R, Greenhalgh T, Harvey G, Walshe K. Realist review: a new method of systematic review designed for complex policy interventions. Journal of health services research & policy. 2005;10(Suppl 1):21-34.
2. Emmel N, Greenhalgh J, Manzano A, Monaghan M, Dalkin S, eds. *Doing realist research*. 2018, Sage: London.
3. Dalkin SM, Greenhalgh J, Jones D, Cunningham B, Lhussier M. What's in a mechanism? Development of a key concept in realist evaluation. Implement Sci. 2015;10:49. 10.1186/s13012-015-0237-x.
4. Jagosh J, Pluye P, Macaulay AC, Salsberg J, Henderson J, Sirett E, et al. Assessing the outcomes of participatory research: protocol for identifying, selecting, appraising and synthesizing the literature for realist review. Implement Sci. 2011;6:24. 10.1186/1748-5908-6-24.
5. Jagosh J, Bush PL, Salsberg J, Macaulay AC, Greenhalgh T, Wong G, et al. A realist evaluation of community-based participatory research: partnership synergy, trust building and related ripple effects. BMC Public Health. 2015;15:725. 10.1186/s12889-015-1949-1.
6. Wong G, Greenhalgh T, Westhorp G, Buckingham J, Pawson R. RAMESES publication standards: realist syntheses. BMC Med. 2013;11:21. 10.1186/1741-7015-11-21.
7. Moher D, Liberati A, Tetzlaff J, Altman DG. The PRISMA Group. Preferred reporting items for systematic reviews and meta-analyses: the PRISMA statement. PLoS Med. 2009;6(7):e10000097.
8. van der Klink JJ, Bultmann U, Burdorf A, Schaufeli WB, Zijlstra FR, Abma FI, et al. Sustainable employability--definition, conceptualization, and implications: A perspective based on the capability approach. Scand J Work Environ Health. 2016;42(1):71-9. 10.5271/sjweh.3531.
9. Sen AK. Development as freedom. New York: Knopf. 1999.
10. Ouzzani M, Hammady H, Fedorowicz Z, Elmagarmid A. Rayyan — a web and mobile app for systematic reviews. Systematic Reviews. 2016;5(210). 10.1186/s13643-016-0384-4.
11. Pluye P, Gagnon MP, Griffiths F, Johnson-Lafleur J. A scoring system for appraising mixed methods research, and concomitantly appraising qualitative, quantitative and mixed methods primary studies in Mixed Studies Reviews. Int J Nurs Stud. 2009;46(4):529-46. 10.1016/j.ijnurstu.2009.01.009.
12. Hong QN, Pluye P, Fàbregues S, Bartlett G, Boardman F, Cargo M, et al. Mixed Methods Appraisal Tool (MMAT), version 2018, Registration of Copyright (#1148552). Canadian Intellectual Property Office, Industry Canada. 2018.
13. Corbiere M, Samson E, Negrini A, St-Arnaud L, Durand MJ, Coutu MF, et al. Factors perceived by employees regarding their sick leave due to depression. Disabil Rehabil. 2016;38(6):511-9. 10.3109/09638288.2015.1046564.
14. Evans-Lacko S, Knapp M. Is manager support related to workplace productivity for people with depression: a secondary analysis of a cross-sectional survey from 15 countries. BMJ Open. 2018;8(6):e021795. 10.1136/bmjopen-2018-021795.
15. Woodall J, Southby K, Trigwell J, Lendzionowski V, Rategh R. Maintaining employment and improving health A qualitative exploration of a job retention programme for employees with mental health conditions. International Journal of Workplace Health Management. 2017;10(1):42-54. 10.1108/Ijwhm-02-2016-0005.
16. Noordik E, Nieuwenhuijsen K, Varekamp I, van der Klink JJ, van Dijk FJ. Exploring the return-to-work process for workers partially returned to work and partially

on long-term sick leave due to common mental disorders: a qualitative study. Disabil Rehabil. 2011;33(17-18):1625-35. 10.3109/09638288.2010.541547.
17. Hammond TE, Crowther A, Drummond S. A Thematic Inquiry into the Burnout Experience of Australian Solo-Practicing Clinical Psychologists. Front Psychol. 2017;8:1996. 10.3389/fpsyg.2017.01996.
18. Chakraborty S, Subramanya AH. Socio-demographic and clinical predictors of absenteeism - A cross-sectional study of urban industrial employees. Ind Psychiatry J. 2013;22(1):17-21. 10.4103/0972-6748.123589.
19. Kok AA, Plaisier I, Smit JH, Penninx BW. The impact of conscientiousness, mastery, and work circumstances on subsequent absenteeism in employees with and without affective disorders. BMC Psychol. 2017;5(1):10. 10.1186/s40359-017-0179-y.
20. Plaisier I, de Graaf R, de Bruijn J, Smit J, van Dyck R, Beekman A, et al. Depressive and anxiety disorders on-the-job: the importance of job characteristics for good work functioning in persons with depressive and anxiety disorders. Psychiatry Res. 2012;200(2-3):382-8. 10.1016/j.psychres.2012.07.016.
21. Laitinen-Krispijn S, Bijl RV. Mental disorders and employee sickness absence: the NEMESIS study. Netherlands Mental Health Survey and Incidence Study. Soc Psychiatry Psychiatr Epidemiol. 2000;35(2):71-7. 10.1007/s001270050010.
22. Richmond MK, Pampel FC, Wood RC, Nunes AP. The impact of employee assistance services on workplace outcomes: Results of a prospective, quasi-experimental study. J Occup Health Psychol. 2017;22(2):170-179. 10.1037/ocp0000018.
23. van den Berg S, Burdorf A, Robroek SJW. Associations between common diseases and work ability and sick leave among health care workers. Int Arch Occup Environ Health. 2017;90(7):685-693. 10.1007/s00420-017-1231-1.
24. Duijts SF, Kant I, van den Brandt PA, Swaen GM. Effectiveness of a preventive coaching intervention for employees at risk for sickness absence due to psychosocial health complaints: results of a randomized controlled trial. J Occup Environ Med. 2008;50(7):765-76. 10.1097/JOM.0b013e3181651584.
25. Leijten FR, van den Heuvel SG, Ybema JF, Robroek SJ, Burdorf A. Do work factors modify the association between chronic health problems and sickness absence among older employees? Scand J Work Environ Health. 2013;39(5):477-85. 10.5271/sjweh.3353.
26. Virtanen M, Vahtera J, Pentti J, Honkonen T, Elovainio M, Kivimaki M. Job strain and psychologic distress influence on sickness absence among Finnish employees. Am J Prev Med. 2007;33(3):182-7. 10.1016/j.amepre.2007.05.003.
27. Lerner D, Adler DA, Rogers WH, Chang H, Lapitsky L, McLaughlin T, et al. Work performance of employees with depression: the impact of work stressors. Am J Health Promot. 2010;24(3):205-13. 10.4278/ajhp.090313-QUAN-103.
28. Hilton MF, Scuffham PA, Sheridan J, Cleary CM, Whiteford HA. Mental ill-health and the differential effect of employee type on absenteeism and presenteeism. J Occup Environ Med. 2008;50(11):1228-43. 10.1097/JOM.0b013e31818c30a8.
29. Loukine L, O'Donnell S, Goldner EM, McRae L, Allen H. Health status, activity limitations, work-related restrictions and level of disability among Canadians with mood and/or anxiety disorders. Health Promot Chronic Dis Prev Can. 2016;36(12):289-301. 10.24095/hpcdp.36.12.03.
30. Keus van de Poll M, Nybergh L, Lornudd C, Hagberg J, Bodin L, Kwak L, et al. Preventing sickness absence among employees with common mental disorders or stress-related symptoms at work: a cluster randomised controlled trial of a problem-solving-based intervention conducted by the Occupational Health Services. Occup Environ Med. 2020;77(7):454-461. 10.1136/oemed-2019-106353.
31. van Mill JG, Vogelzangs N, Hoogendijk WJ, Penninx BW. Sleep disturbances and reduced work functioning in depressive or anxiety disorders. Sleep Med. 2013;14(11):1170-7. 10.1016/j.sleep.2013.04.016.
32. Woo JM, Kim W, Hwang TY, Frick KD, Choi BH, Seo YJ, et al. Impact of depression on work productivity and its improvement after outpatient treatment with antidepressants. Value Health. 2011;14(4):475-82. 10.1016/j.jval.2010.11.006.
33. Ridge D, Broom A, Kokanovic R, Ziebland S, Hill N. Depression at work, authenticity in question: Experiencing, concealing and revealing. Health (London). 2019;23(3):344-361. 10.1177/1363459317739437.
34. Mackenzie A, Harvey S, Mewton L, Andrews G. Occupational impact of internet-delivered cognitive behaviour therapy for depression and anxiety: reanalysis of data from five Australian randomised controlled trials. Med J Aust. 2014;201(7):417-9. 10.5694/mja14.00293.
35. Rost K, Smith JL, Dickinson M. The effect of improving primary care depression management on employee absenteeism and productivity. A randomized trial. Med

Care. 2004;42(12):1202-10. 10.1097/00005650-200412000-00007.
36. Sahlin E, Ahlborg G, Jr., Matuszczyk JV, Grahn P. Nature-based stress management course for individuals at risk of adverse health effects from work-related stress-effects on stress related symptoms, workability and sick leave. Int J Environ Res Public Health. 2014;11(6):6586-611. 10.3390/ijerph110606586.
37. Arends I, Almansa J, Stansfeld SA, Amick BC, van der Klink JJL, Bultmann U. One-year trajectories of mental health and work outcomes post return to work in patients with common mental disorders. J Affect Disord. 2019;257:263-270. 10.1016/j.jad.2019.07.018.
38. Danielsson L, Elf M, Hensing G. Strategies to keep working among workers with common mental disorders - a grounded theory study. Disabil Rehabil. 2017;41(7):786-795. 10.1080/09638288.2017.1408711.
39. Linden M, Zubragel D, Bar T. Occupational functioning, sickness absence and medication utilization before and after cognitive-behaviour therapy for generalized anxiety disorders. Clin Psychol Psychother. 2011;18(3):218-24. 10.1002/cpp.712.
40. Ebert DD, Heber E, Berking M, Riper H, Cuijpers P, Funk B, et al. Self-guided internet-based and mobile-based stress management for employees: results of a randomised controlled trial. Occup Environ Med. 2016;73(5):315-23. 10.1136/oemed-2015-103269.
41. Birney AJ, Gunn R, Russell JK, Ary DV. MoodHacker Mobile Web App With Email for Adults to Self-Manage Mild-to-Moderate Depression: Randomized Controlled Trial. JMIR Mhealth Uhealth. 2016;4(1):e8. 10.2196/mhealth.4231.
42. Wang PS, Simon GE, Avorn J, Azocar F, Ludman EJ, McCulloch J, et al. Telephone screening, outreach, and care management for depressed workers and impact on clinical and work productivity outcomes: a randomized controlled trial. JAMA. 2007;298(12):1401-11. 10.1001/jama.298.12.1401.
43. Vlasveld MC, van der Feltz-Cornelis CM, Anema JR, van Mechelen W, Beekman AT, van Marwijk HW, et al. The associations between personality characteristics and absenteeism: a cross-sectional study in workers with and without depressive and anxiety disorders. J Occup Rehabil. 2013;23(3):309-17. 10.1007/s10926-012-9406-9.
44. Kawakami N, Haratani T, Iwata N, Imanaka Y, Murata K, Araki S. Effects of mailed advice on stress reduction among employees in Japan: a randomized controlled trial. Ind Health. 1999;37(2):237-42. 10.2486/indhealth.37.237.
45. Telle NT, Moock J, Heuchert S, Schulte V, Rossler W, Kawohl W. Job Maintenance through Supported Employment PLUS: A Randomized Controlled Trial. Front Public Health. 2016;4:194. 10.3389/fpubh.2016.00194.
46. Lexis MA, Jansen NW, Huibers MJ, van Amelsvoort LG, Berkouwer A, Tjin ATG, et al. Prevention of long-term sickness absence and major depression in high-risk employees: a randomised controlled trial. Occup Environ Med. 2011;68(6):400-7. 10.1136/oem.2010.057877.
47. Cocker F, Martin A, Scott J, Venn A, Otahal P, Sanderson K. Factors associated with presenteeism among employed Australian adults reporting lifetime major depression with 12-month symptoms. J Affect Disord. 2011;135(1-3):231-40. 10.1016/j.jad.2011.07.028.
48. Uribe JM, Pinto DM, Vecino-Ortiz AI, Gomez-Restrepo C, Rondon M. Presenteeism, Absenteeism, and Lost Work Productivity among Depressive Patients from Five Cities of Colombia. Value Health Reg Issues. 2017;14:15-19. 10.1016/j.vhri.2017.03.001.
49. Lexis MA, Jansen NW, van Amelsvoort LG, van den Brandt PA, Kant I. Depressive complaints as a predictor of sickness absence among the working population. J Occup Environ Med. 2009;51(8):887-95. 10.1097/JOM.0b013e3181aa012a.
50. Plaisier I, Beekman AT, de Graaf R, Smit JH, van Dyck R, Penninx BW. Work functioning in persons with depressive and anxiety disorders: the role of specific psychopathological characteristics. J Affect Disord. 2010;125(1-3):198-206. 10.1016/j.jad.2010.01.072.
51. Daley M, Morin CM, LeBlanc M, Gregoire JP, Savard J, Baillargeon L. Insomnia and its relationship to health-care utilization, work absenteeism, productivity and accidents. Sleep Med. 2009;10(4):427-38. 10.1016/j.sleep.2008.04.005.
52. Sado M, Shirahase J, Yoshimura K, Miura Y, Yamamoto K, Tabuchi H, et al. Predictors of repeated sick leave in the workplace because of mental disorders. Neuropsychiatr Dis Treat. 2014;10:193-200. 10.2147/NDT.S55490.
53. Dunner DL, Kwong WJ, Houser TL, Richard NE, Donahue RM, Khan ZM. Improved Health-Related Quality of Life and Reduced Productivity Loss After Treatment With Bupropion Sustained Release: A Study in Patients With Major Depression. Prim Care Companion J Clin Psychiatry. 2001;3(1):10-16. 10.4088/pcc.v03n0103.

54. Johnson JR, Emmons HC, Rivard RL, Griffin KH, Dusek JA. Resilience Training: A Pilot Study of a Mindfulness-Based Program with Depressed Healthcare Professionals. Explore (NY). 2015;11(6):433-44. 10.1016/j.explore.2015.08.002.
55. O'Haire ME, Rodriguez KE. Preliminary efficacy of service dogs as a complementary treatment for posttraumatic stress disorder in military members and veterans. J Consult Clin Psychol. 2018;86(2):179-188. 10.1037/ccp0000267.
56. Swanson LM, Arnedt JT, Rosekind MR, Belenky G, Balkin TJ, Drake C. Sleep disorders and work performance: findings from the 2008 National Sleep Foundation Sleep in America poll. J Sleep Res. 2011;20(3):487-94. 10.1111/j.1365-2869.2010.00890.x.
57. Jha MK, Minhajuddin A, Greer TL, Carmody T, Rush AJ, Trivedi MH. Early Improvement in Work Productivity Predicts Future Clinical Course in Depressed Outpatients: Findings From the CO-MED Trial. Am J Psychiatry. 2016;173(12):1196-1204. 10.1176/appi.ajp.2016.16020176.
58. Chen SW, Wang PC, Hsin PL, Oates A, Sun IW, Liu SI. Job stress models, depressive disorders and work performance of engineers in microelectronics industry. Int Arch Occup Environ Health. 2011;84(1):91-103. 10.1007/s00420-010-0538-y.
59. Lerner D, Adler DA, Rogers WH, Ingram E, Oslin DW. Effect of Adding a Work-Focused Intervention to Integrated Care for Depression in the Veterans Health Administration: A Randomized Clinical Trial. JAMA Netw Open. 2020;3(2):e200075. 10.1001/jamanetworkopen.2020.0075.
60. Haslam C, Atkinson S, Brown SS, Haslam RA. Anxiety and depression in the workplace: effects on the individual and organisation (a focus group investigation). J Affect Disord. 2005;88(2):209-15. 10.1016/j.jad.2005.07.009.
61. Adler DA, McLaughlin TJ, Rogers WH, Chang H, Lapitsky L, Lerner D. Job performance deficits due to depression. Am J Psychiatry. 2006;163(9):1569-76. 10.1176/ajp.2006.163.9.1569.
62. Petersson EL, Wikberg C, Westman J, Ariai N, Nejati S, Bjorkelund C. Effects on work ability, job strain and quality of life of monitoring depression using a self-assessment instrument in recurrent general practitioner consultations: A randomized controlled study. Work. 2018;60(1):63-73. 10.3233/WOR-182717.
63. Rothermund E, Gundel H, Rottler E, Holzer M, Mayer D, Rieger M, et al. Effectiveness of psychotherapeutic consultation in the workplace: a controlled observational trial. BMC Public Health. 2016;16:891. 10.1186/s12889-016-3567-y.
64. Kim J, Kim YK, Leem SH, Won JU. Association between job-related stress and experience of presenteeism among Korean workers stratified on the presence of depression. Ann Occup Environ Med. 2019;31:e26. 10.35371/aoem.2019.31.e26.
65. Bertilsson M, Petersson EL, Ostlund G, Waern M, Hensing G. Capacity to work while depressed and anxious--a phenomenological study. Disabil Rehabil. 2013;35(20):1705-11. 10.3109/09638288.2012.751135.
66. Furukawa TA, Horikoshi M, Kawakami N, Kadota M, Sasaki M, Sekiya Y, et al. Telephone cognitive-behavioral therapy for subthreshold depression and presenteeism in workplace: a randomized controlled trial. PLoS One. 2012;7(4):e35330. 10.1371/journal.pone.0035330.
67. Lindsater E, Axelsson E, Salomonsson S, Santoft F, Ejeby K, Ljotsson B, et al. Internet-Based Cognitive Behavioral Therapy for Chronic Stress: A Randomized Controlled Trial. Psychother Psychosom. 2018;87(5):296-305. 10.1159/000490742.
68. Lappalainen P, Kaipainen K, Lappalainen R, Hoffren H, Myllymaki T, Kinnunen ML, et al. Feasibility of a personal health technology-based psychological intervention for men with stress and mood problems: randomized controlled pilot trial. JMIR Res Protoc. 2013;2(1):e1. 10.2196/resprot.2389.
69. Lam RW, Lutz K, Preece M, Cayley PM, Bowen Walker A. Telephone-administered cognitive-behavioral therapy for clients with depressive symptoms in an employee assistance program: a pilot study. Ann Clin Psychiatry. 2011;23(1):11-6.
70. Beck A, Crain LA, Solberg LI, Unutzer J, Maciosek MV, Whitebird RR, et al. The effect of depression treatment on work productivity. Am J Manag Care. 2014;20(8):e294-301.
71. Nigatu YT, Reijneveld SA, Penninx BW, Schoevers RA, Bultmann U. The longitudinal joint effect of obesity and major depression on work performance impairment. Am J Public Health. 2015;105(5):e80-6. 10.2105/AJPH.2015.302557.
72. Okajima I, Akitomi J, Kajiyama I, Ishii M, Murakami H, Yamaguchi M. Effects of a Tailored Brief Behavioral Therapy Application on Insomnia Severity and Social Disabilities Among Workers With Insomnia in Japan: A Randomized Clinical Trial. JAMA Netw Open. 2020;3(4):e202775. 10.1001/jamanetworkopen.2020.2775.
73. Danielsson L, Waern M, Hensing G, Holmgren K. Work-directed rehabilitation or physical activity to support work ability and mental health in common mental

disorders: a pilot randomized controlled trial. Clin Rehabil. 2020;34(2):170-181. 10.1177/0269215519880230.

Appendix B

Example search string

Pubmed 26-6-2020

#1 "Mental Disorders"[Mesh:NoExp] OR Mentally Ill Persons[MH] OR Anxiety disorders[MH] OR anxiety[MH] OR Agoraphobia[MH] OR Panic disorder[MH] OR Obsessive-compulsive disorder[MH] OR Mood disorders[MH] OR Depression[MH] OR Depressive disorder[MH] OR Phobia, social[MH] OR Somatoform disorders[MH] OR Adjustment disorders[MH] OR Burnout, professional[MH] OR Trauma and Stressor Related Disorders[MH] OR Mental disorder*[TIAB] OR Psychiatric diagnosis[TIAB] OR Mentally Ill[TIAB] OR Mental patients[TIAB] OR anxiety disorder*[TIAB] OR panic disorders[TIAB] OR panic attack*[TIAB] OR social phobia*[TIAB] OR social anxiety disorder*[TIAB] OR agoraphobias[TIAB] OR obsessive compulsive disorder[TIAB] OR anankastic personality[TIAB] OR Mood disorder[TIAB] OR affective disorder*[TIAB] OR depression*[TIAB] OR depressive symptom[TIAB] OR emotional depression*[TIAB] OR depressive syndrome[TIAB] OR unipolar depression[TIAB] OR somatization disorder[TIAB] OR somatoform disorder[TIAB] OR reactive disorder*[TIAB] OR adjustment disorder[TIAB] OR reactive depression[TIAB] OR Professional burnout[TIAB] OR occupational burnout[TIAB] OR traumatic stress disorder*[TIAB] OR dysthymic disorders[TIAB] OR Common mental health*[TIAB] OR Common mental disorder*[TIAB] OR Common mental ill*[TIAB] OR Common mental diseas*[TIAB] OR Common psychiatric disorder*[TIAB] OR Anxiety disorder*[TIAB] OR generalized anxiet*[TIAB] OR Agoraphobia*[TIAB] OR Panic disorder*[TIAB] OR OCD[TIAB] OR Mood Disorder*[TIAB] OR Affective Disorder*[TIAB] OR reactive depression*[TIAB] OR recurrent depression*[TIAB] OR depressive disorder*[TIAB] OR Simple phobia*[TIAB] OR Social phobia*[TIAB] OR Burnout[TIAB] OR Burn out[TIAB] OR Somatoform disorder*[TIAB] OR Adjustment disorder*[TIAB] OR Mental health disabilit*[TIAB] OR Acute stress disorder[TIAB]

749,706

#2 "Employment"[Mesh] OR "Work"[Mesh] OR Employe*[tiab] OR Employment*[tiab] OR Labour*[tiab] OR Labor*[tiab] OR Worker*[tiab] OR Occupation*[tiab] OR vocational*[tiab]

1,456,306

#3 stress related symptom*[TIAB] OR Nervousness*[TIAB] OR Tension*[TIAB] OR worry symptoms[TIAB] OR irritability*[TIAB] OR mental fatigue*[TIAB] OR tiredness[TIAB] OR insomnia[TIAB] OR headache*[TIAB] OR sadness[TIAB] OR concentration[TIAB] OR restlessness[TIAB] OR sleeplessness[TIAB] OR depressed mood*[TIAB] OR fear[TIAB] OR ambivalence[TIAB] OR negative emotion*[TIAB] OR negative feeling*[tiab] OR marked distress[TIAB] OR psychological distress[TIAB] OR exhaustion[TIAB] OR workaholism[TIAB] OR Work related strain[TIAB] OR Work related sleep disturbances[TIAB]

1,520,404

#4 #2 AND #3

102,700

#5 #1 OR #4

845,205

#6 Stay at work*[TIAB] OR Staying at work[TIAB] OR Job retention*[TIAB] OR Job tenur*[TIAB] OR Sustainable employment*[TIAB] OR Sustainable employability[TIAB] OR Presenteeism[TIAB] OR "continue work*"[TIAB] OR continue working[TIAB] OR ongoing employment[TIAB] OR (Partial*[tiab] AND (returned to work[tiab] OR return to work[tiab])) OR partial sick leave*[tiab] OR partial absenteeism[tiab] OR improved work ability[tiab] OR job maintenance[TIAB] OR work maintenance[TIAB] OR partial absenteeism[TIAB] OR partial sick leave*[TIAB]

2727

#7 prevention and control [Subheading] OR reduc*[tiab] OR prevent*[TIAB]

5,113,293

#8 Absenteeism[MH] OR sick leave[MH] OR Absenteeism[TIAB] OR Work absence[TIAB] OR Sickness absen*[TIAB] OR Job absen*[TIAB] OR Work disability[TIAB] OR sick leave*[TIAB] OR Work loss[TIAB] OR Job loss[TIAB] OR Occupational loss[TIAB] OR Occupational disease[TIAB] OR Occupational illness[TIAB] OR Presenteeism[MH] OR underemployment[tiab] OR under employ*[tiab] OR underemploy*[tiab] OR Disability leave*[TIAB] OR sick day*[TIAB] OR Illness day*[TIAB] OR workplace productivit*[tiab] OR work impairment[tiab] OR work performance*[tiab] OR work participation[tiab] OR work functioning[tiab] OR work abilit*[TIAB]

30593

#9 #7 AND #8

9933

#10 #6 OR #9

12087

Stap 1:

#11 #5 AND #6

571

Stap 2:

#12 #5 AND #10

2186

#13 medline[sb]

25180969

#14 #11 NOT #13

97

#15 #12 NOT #13

269

Publication date from 1995/01/01

Totaal: 268

----- end ------
